# Metabolomics Analysis Across Multiple Biofluids Reveals the Metabolic Responses of Lactating Holstein Dairy Cows to Fermented Soybean Meal Replacement

**DOI:** 10.3389/fvets.2022.812373

**Published:** 2022-05-13

**Authors:** Zuo Wang, Yuannian Yu, Weijun Shen, Zhiliang Tan, Shaoxun Tang, Hui Yao, Jianhua He, Fachun Wan

**Affiliations:** ^1^College of Animal Science and Technology, Hunan Agricultural University, Changsha, China; ^2^Rudong Agriculture Bureau, Nantong, China; ^3^CAS Key Laboratory of Agro-Ecological Processes in Subtropical Region, National Engineering Laboratory for Pollution Control and Waste Utilization in Livestock and Poultry Production, Hunan Provincial Key Laboratory of Animal Nutrition Physiology and Metabolism, Institute of Subtropical Agriculture, Chinese Academy of Sciences, Changsha, China; ^4^Nanshan Dairy Co., Ltd., Shaoyang, China

**Keywords:** dairy cow, fermented soybean meal, metabolite, metabolomics, multiple biofluids

## Abstract

This experiment was performed to reveal the metabolic responses of dairy cows to the replacement of soybean meal (SBM) with fermented soybean meal (FSBM). Twenty-four lactating Chinese Holstein dairy cattle were assigned to either the SBM group [the basal total mixed ration (TMR) diet containing 5.77% SBM] or the FSBM group (the experimental TMR diet containing 5.55% FSBM), in a completely randomized design. The entire period of this trial consisted of 14 days for the adjustment and 40 days for data and sample collection, and sampling for rumen liquid, blood, milk, and urine was conducted on the 34th and 54th day, respectively. When SBM was completely replaced by FSBM, the levels of several medium-chain FA in milk (i.e., C13:0, C14:1, and C16:0) rose significantly (*p* < 0.05), while the concentrations of a few milk long-chain FA (i.e., C17:0, C18:0, C18:1n9c, and C20:0) declined significantly (*p* < 0.05). Besides, the densities of urea nitrogen and lactic acid were significantly (*p* < 0.05) higher, while the glucose concentration was significantly (*p* < 0.05) lower in the blood of the FSBM-fed cows than in the SBM-fed cows. Based on the metabolomics analysis simultaneously targeting the rumen liquid, plasma, milk, and urine, it was noticed that substituting FSBM for SBM altered the metabolic profiles of all the four biofluids. According to the identified significantly different metabolites, 3 and 2 amino acid-relevant metabolic pathways were identified as the significantly different pathways between the two treatments in the rumen fluid and urine, respectively. Furthermore, glycine, serine, and threonine metabolism, valine, leucine, and isoleucine biosynthesis, and cysteine and methionine metabolism were the three key integrated different pathways identified in this study. Results mainly implied that the FSBM replacement could enhance nitrogen utilization and possibly influence the inflammatory reactions and antioxidative functions of dairy cattle. The differential metabolites and relevant pathways discovered in this experiment could serve as biomarkers for the alterations in protein feed and nitrogen utilization efficiency of dairy cows, and further investigations are needed to elucidate the definite roles and correlations of the differential metabolites and pathways.

## Introduction

Protein is an indispensable component in the ration for dairy cows, it offers rumen microbe nitrogen and amino acids to synthesize the microbial protein, as well as meets the nutritional demands for diverse metabolisms of the animal (1, 2). As the most commonly adopted protein source for dairy cattle, soybean meal milk (SBM) could be characterized by its well-balanced amino acid profile, bountiful rumen degradable protein (RDP) amount, and high digestibility of cellulose and pectin (1, 3). Rego et al. (4) reported that dietary inclusion of SBM enhanced the dry matter intake, milk yield, and milk protein production of grazing dairy cattle having *ad libitum* access to grass silage.

Nevertheless, the nutritional drawbacks of SBM have also been noticed, which are mainly reflected by the low rumen undegradable protein (RUP) content and the existence of various anti-nutritional factors (e.g., trypsin inhibitors, haemagglutinins, raffinose, and stachyose) (1, 5, 6). The promotion of the overall quality of SBM can be achieved via the microbial fermentation process (7). In the fermented soybean meal (FSBM), the concentration of RUP could be raised by the heat treatment during the fermentation processing (8). Moreover, microbial fermentation has been verified to reduce or remove the anti-nutritional agents in SBM (9, 10). In addition, the concentrations of non-protein nitrogen (e.g., small peptides, free amino acids, and ammonia) and vitamins would be increased through fermentation (3, 11).

By far, the majority of the research targeting the effects of FSBM on dairy cattle has been performed on calves. It was observed that feeding FSBM relieved the weaning stress and improved the immune status of weaned and lipopolysaccharide (LPS)-challenged calves, since the FSBM supplementation led to greater concentrations of LPS-specific IgG, LPS-specific IgA, and haptoglobin, but a lower level of cortisol in the peripheral blood (12). As reported by Rezazadeh et al. (13), not only the alleviated weaning stress resulting from the declines in pro-inflammatory mediators but also the enhanced growth performance was presented by the FSBM-fed calves when compared to the SBM-fed calves during cold weather. More recently, it was found that the replacement of SBM with FSBM could boost the calf performance by manipulating rumen fermentation and the ruminal bacterial community (11). However, information on the influences of feeding FSBM on lactating cows remains rather limited. As revealed by our precedent study (14), the substitution of FSBM for SBM modulated the rumen fermentation and rumen bacterial microflora in lactating Holstein dairy cows. Therefore, based on the significance of rumen microbial fermentation in ruminant metabolisms (15), it could be hypothesized that replacing SBM with FSBM could possibly exert influences on the metabolisms of lactating dairy cows.

Metabolomics is a systemic biological approach to investigating the overall metabolism responding to the internal or external alterations by identifying and quantifying the endogenous small molecular metabolites in the physiological fluids and tissues (16, 17). During the past few years, by using a high-throughput method, metabolomics analysis has been rapidly applied in the studies on profiling the metabolism mechanisms of dairy cows related to varying animal performances, diets, diseases, and environments (17–23). It is noteworthy that amongst different metabolomics approaches, the multiple biofluid metabolomics provides insights into the metabolic biomarkers, pathways, and metabolism patterns by simultaneously taking different biofluids (e.g., rumen liquid, blood, milk, and urine) into consideration, which could lead to a better understanding on the comprehensive endogenous metabolic condition of dairy cattle (17, 18, 22).

In the current study, we aimed to evaluate the effects of replacing SBM with FSBM in the diets of lactating Holstein dairy cows through non-targeted metabolomics analysis across four biofluids (i.e., rumen liquid, plasma, milk, and urine) based on the liquid chromatography-mass spectrometry (LC-MS) platform, together with assessing indices on milk fatty acids (FA) composition, blood physiology, and biochemistry. The purpose of this investigation was to gain insights into the metabolomes of different biofluids, and their correlations and responses to the substitution of FSBM for SBM, so as to provide the application of dietary FSBM in lactating ruminants with better references.

## Materials and Methods

### Experimental Designs, Diets, and Management

All procedures involving animals in the present experiment were approved by the Animal Care Committee (approval number: 20190602), College of Animal Science and Technology, Hunan Agricultural University, Changsha, China. This trial was performed at the Nanshan Dairy Farm (Shaoyang, Hunan Province, China). Twenty-four lactating Chinese Holstein dairy cows averaging (initial mean ± SD) 20 ± 3.4 kg of milk/day, 164 ± 46 days in milk, 2 ± 1 of parity, and 460 ± 50 kg of body weight were used as the experimental animals in a completely randomized design. Cattle were randomly assigned to either the SBM group (the basal TMR diet containing 5.77% SBM) or the FSBM group (the test TMR diet containing 5.55% FSBM). The FSBM used in this trial was a commercial product and fermented with the inoculation of *Lactobacillus* spp., *Bacillus subtilis*, and *Saccharomyces cerevisae* (Minxiong Biotech. Co., Ltd., Longyan, China). The nutritional compositions of the SBM and FSBM are exhibited in Supplementary Table 1, while the ingredients and nutrient contents of the two diets are displayed in Table 1. The whole experimental period was 54 days, comprising 14 days of adjustment and 40 days of data and sample collection. All cattle were housed in a tie-stall barn and fed *ad libitum* twice per day at 06:00 and 18:00 h with free access to fresh water.

**Table 1:** Ingredients and chemical composition of rations for the SBM group and FSBM group.

	**SBM^a^**	**FSBM^b^**
**Ingredients, % DM**		
Corn	17.68	17.89
Wheat flour	4.7	4.7
Corn germ meal	1.75	2.44
DDGS^c^	6.41	5.77
Sprayed corn bran	5.13	5.13
Soybean meal	5.77	–
Fermented soybean meal	–	5.55
Alfalfa grass	8.62	8.62
Oat grass	4.42	4.42
*Leymus chinensis* hay	4.44	4.44
Corn silage	33.36	33.36
Whole cottonseed	5.95	5.95
Urea	0.04	–
NaCl	0.27	0.27
CaHPO_4_	0.44	0.44
CaCO_3_	0.56	0.56
Premix^d^	0.46	0.46
**Chemical composition, % DM**		
NE_L_, Mcal/kg	1.61	1.61
Organic matter	91.50	91.40
Crude protein	15.87	15.92
Neutral detergent fiber	35.50	36.00
Acid detergent fiber	22.81	22.95
Ether extract	4.33	4.33
Ash	8.50	8.60
Ca	0.51	0.52
P	0.39	0.39

a *SBM, soybean meal*;

b *FSBM, fermented soybean meal*;

c *DDGS, distillers' dried grains with soluble*;

d *Every 1 kg of premix contained 400 mg of Zn, 100 mg of Cu, 200 mg of Fe, 3,600 mg of Mg, 350 mg of Mu, 96 mg of Cr, 4.0 mg of Co, 50 mg of Se, 500 mg of Lysine, 500 mg of Methionine, 25,00,000 IU of vitamin A, 100,000 IU of vitamin D3, and 4,000 IU of vitamin E*.

### Sample Collection

The collection of samples was conducted on 34 and 54 days of the present experiment, respectively. The rumen fluid from the central rumen of each cow was, respectively, collected 2 h before and 4 h after morning feeding, using an oral stomach tube through the oral cavity as described previously (24). In short, the initial 150 ml of rumen fluid was discarded, before the following 150 ml was collected and further filtered using four layers of gauzes under a continuous CO_2_ stream. The rumen liquid samples obtained at the above two time points were pooled at the ratio of 1:1. Blood was collected into evacuated tubes via the tail vein of each cow before the morning feeding and then placed at room temperature for 30 min, followed by centrifugation at 1,500 × g for 10 min at 4°C to attain the serum samples for the analysis of serum physiology and biochemistry. As for the plasma samples, blood was firstly acquired in a 10 ml anti-coagulation (heparin sodium) tube and subsequently centrifuged at 3,500 × g for 15 min at 4°C. The milk samples were firstly collected, respectively, since the cattle were milked twice (08:30 and 20:00 h) per day and then mixed together at the ratio of 1:1, based on the proportion between each milking yield. To obtain the supernatant urine samples, 50 ml of the mid-stream urine was collected into a plastic tube during the urination of each cow before morning feeding, followed by centrifugation at 2,000 × g for 5 min. For the metabolomics analysis, six cows from each treatment were randomly selected and their biofluid samples (rumen liquid, plasma, milk, and urine) collected on 34 and 54 days were further separately pooled at the ratio of 1:1. All samples were immediately frozen in liquid nitrogen and then stored at −80°C until further analysis.

### Chemical and Biochemical Analysis

The dry matter (DM; method 930.15), ash (method 942.05), crude protein (method 2001.11), ether extract (method 920.39), neutral detergent fiber (NDF; method 2002.04), and acid detergent fiber (ADF; method 973.18) of the SBM, FSBM, and the two diets were analyzed according to the procedures of AOAC (25). The contents of calcium (Ca) and phosphorus (P) in the SBM, FSBM, and the two rations were measured as previously depicted (26, 27).

The compositions of FA in the milk were measured according to the procedures in earlier studies (28–30). The milk samples were firstly methylated to prepare the fatty acid methyl esters (FAME), and the FAME recovered by hexane were then analyzed using a gas chromatograph (HP7890A, Agilent Technologies, Santa Clara, USA) equipped with a flame-ionization detector. The identification of peaks was achieved by comparison of the retention times with FAME standards (18919-1AMP, Sigma Aldrich, Saint Louis, USA). The peak integration and analytes assessment were operated with the Chemstation software (Agilent Technologies, Santa Clara, USA).

For the assessments of those biochemical blood indices displayed in **Table 3**, a Roche Cobas automatic biochemistry analyzer (c311, Roche Ltd., Basel, Switzerland) and related specific kits (Roche Ltd., Basel, Switzerland) were applied, according to the manufacturer's instructions and prior research (31).

### Metabolites Extraction

The preparation of the collected biofluid samples (rumen liquid, plasma, milk, and urine) for ultra-high performance liquid chromatography-mass spectrometry (UHPLC-MS) analysis was performed by referring to the precedently described procedures (17) with modifications. First, 400 μl of extract solution (acetonitrile: methanol = 1:1) containing the isotopically-labeled internal standard mixture was added to 100 μl of biofluid sample. After the successive vortex for 30 s, sonication for 10 min in ice–water bath, and incubation at −20°C for 1 h, the samples were further centrifuged at 12,000 rpm for 15 min at 4°C. The final supernatant was transferred to a fresh glass vial for analysis. The quality control (QC) sample was prepared by mixing an equal aliquot of the supernatants from all of the samples.

### UHPLC-MS Analysis

UHPLC-MS analysis was conducted through a Vanquish UHPLC system (Thermo Fisher Scientific, Waltham, USA) with a UPLC BEH Amide column (2.1 mm × 100 mm, 1.7 μm) coupled to an Orbitrap Q Exactive HFX mass spectrometer (Thermo Fisher Scientific, Waltham, USA) by the Biotree Biomed. Tech. Co., Ltd. (Shanghai, China). The mobile phase consisted of 25 mM ammonium acetate and 25 mM ammonia hydroxide in water (pH = 9.75) (eluent A) and acetonitrile (eluent B). The analysis was carried out with elution gradient set as follows: 0–0.5 min, 95% B; 0.5–7.0 min, 95–65% B; 7.0–8.0 min, 65–40% B; 8.0–9.0 min, 40% B; 9.0–9.1 min, 40–95% B; 9.1–12.0 min, 95% B. The column temperature and the auto-sampler temperature were, respectively, 25 and 4°C, and the injection volume was 2 μl.

The HFX mass spectrometer was used to obtain MS/MS spectra in the information-dependent acquisition (IDA) mode in the control of the Xcalibur acquisition software (Thermo Fisher Scientific, Waltham, USA). In this mode, the acquisition software continuously evaluates the full scan MS spectrum. The ESI source conditions were, respectively, set as follows: sheath gas flow rate as 50 Arb, Aux gas flow rate as 10 Arb, capillary temperature at 320°C, full MS resolution as 60000, MS/MS resolution as 7500, collision energy as 10/30/60 in NCE mode, spray voltage as 3.5 kV (positive polarity mode) or −3.2 kV (negative polarity mode).

### Data Processing and Metabolite Annotation

The raw data generated during the UHPLC-MS/MS analysis were converted to the mzXML format using ProteoWizard (version 3.0.9134) and processed with an in-house program, which was developed using R (version 3.4.3) and based on the R script XCMS, for peak detection, extraction, alignment, and integration. Afterward, an in-house MS2 commercial database (Biotree Biomed. Tech. Co., Ltd, Shanghai, China) was employed in metabolite annotation, using the R page CAMERA for collection of annotation relevant methods from MS data (32). The similarity cut-off for annotation was set at 0.3. The measurements were not counted when detected peaks were less than half of QC samples or the standard deviation is above 30%.

### Statistical and Data Analysis

The PROC MIXED procedure of SAS (version 9.4, SAS Institute Inc.) was used to examine the effects of substituting FSBM for SBM on the parameters for milk composition, blood physiology, and biochemistry. The statistical model included treatment and sampling date as the fixed effects, sampling date as repeated determination, and animal as the random effect. Least squares means were presented throughout the text. Statistical difference was, respectively, declared as significant or highly significant at *p* < 0.05 or *p* < 0.01, while trend was discussed at 0.05 < *p* ≤ 0.10.

For the metabolomics data analysis, metabolites identified under the positive and negative polarity mode in this study were both combined to unify the data. After the internal standard normalization and subsequent logarithmic transformation of the data, the principal components analysis (PCA) and orthogonal projections to latent structures-discriminant analysis (OPLS-DA) were conducted through the SIMCA software (version 15.0.2, Sartorius Stedim Data Analytics AB, Umea, Sweden). The one-way ANOVA was adopted to analyze the significant change of each metabolite between different treatments. The differential metabolites were classified through two-group comparisons and the significance was determined using *t*-test. Metabolites with the variable importance in projection (VIP) value > 1 and *p* < 0.05 were considered as the significantly differential metabolites. The metabolic pathway and enrichment analysis were operated using MetaboAnalyst (version 4.0) based on the Kyoto encyclopedia of genes and genomes (KEGG) database (33, 34).

## Results

### Changes in the Milk FA Composition by FSBM Replacement

The proportions of C13:0 (*p* < 0.05), C14:1 (*p* < 0.01), and C16:0 (*p* < 0.01) in the milk of dairy cattle rose significantly in response to the FSBM replacement (Table 2). In contrast, the ratios of C17:0 (*p* < 0.05), C18:0 (*p* < 0.01), and C18:1n9c (*p* < 0.05) were significantly reduced by the substitution of FSBM for SBM. Moreover, it is noted that the FSBM replacement tended to raise the percentage of C14:0 (*p* < 0.1). The remaining FA and the sums of both saturated FA and unsaturated FA were unaffected (*p* > 0.05) when the SBM was replaced by FSBM in the diet of dairy cows.

**Table 2:** Comparison of milk FA proportions (g/100 g of FA) between the SBM group and FSBM group.

**Fatty acid**	**Treatment**		**SEM^3^**	***p*-Value**
	**SBM^1^**	**FSBM^2^**		
C4:0	2.35	2.52	0.125	0.311
C6:0	3.08	3.32	0.137	0.205
C8:0	2.35	2.37	0.070	0.856
C10:0	5.05	4.87	0.160	0.403
C12:0	4.77	4.67	0.153	0.641
C13:0	0.17^b^	0.20^a^	0.009	0.037
C14:0	12.54	13.11	0.243	0.099
C14:1	0.98^b^	1.20^a^	0.058	0.009
C15:0	1.22	1.26	0.028	0.283
C16:0	28.9^b^	31.33^a^	0.522	0.002
C16:1	1.57	1.66	0.081	0.463
C17:0	0.72^a^	0.67^b^	0.016	0.017
C18:0	10.69^a^	9.05^b^	0.399	0.006
C18:1n9c	21.42^a^	19.55^b^	0.529	0.015
C18:1n9t	0.27	0.28	0.011	0.627
C18:2n6c	2.90	2.78	0.095	0.356
C18:3n3	0.42	0.41	0.015	0.730
C20:0	0.14^a^	0.13^b^	0.005	0.025
C20:3n6	0.16	0.14	0.008	0.271
C20:4n6	0.28	0.28	0.010	0.900
Σ SFA^4^	72.25	73.68	0.685	0.135
Σ UFA^5^	27.65	26.22	0.683	0.136

a,b *Means within a row for treatments that do not have a common superscript differ*.

1 *SBM, soybean meal*;

2 *FSBM, fermented soybean meal*;

3 *SEM for treatments*;

4 *Sum of saturated fatty acids*;

5 *Sum of unsaturared fatty acids*.

### Changes in the Biochemical Blood Indices by FSBM Replacement

The FSBM replacement significantly increased the concentrations of urea nitrogen (*p* < 0.05) and lactic acid (*p* < 0.05), and it also tended to enhance the amount of uric acid (*p* < 0.1) in the blood of dairy cattle (Table 3). Nonetheless, the level of glucose significantly declined (*p* < 0.01) as FSBM was substituted for SBM. No significant difference (*p* > 0.05) was observed in other biochemical blood parameters between the SBM and FSBM treatments.

**Table 3:** Comparison of specific biochemical blood parameters between the SBM group and FSBM group.

**Item**	**Treatment**		**SEM^3^**	***p*-Value**
	**SBM^1^**	**FSBM^2^**		
Total proteins, g/L	75.6	77.7	1.66	0.219
Albumin, g/L	33.1	33.3	0.57	0.383
Globulin, g/L	41.9	43.3	1.45	0.431
Urea nitrogen, mmol/L	4.03^b^	4.46^a^	0.124	0.013
Uric acid, mmol/L	0.70	0.76	0.025	0.098
Ammonia, μmol/L	126.7	131.2	4.07	0.418
Creatinine, μmol/L	64.7	65.0	2.23	0.918
Glucose, mmol/L	2.91^a^	2.63^b^	0.113	0.003
Triglycerides, mmol/L	0.17	0.17	0.009	0.915
Cholesterol, mmol/L	3.47	3.46	0.161	0.974
High-density lipoprotein, mmol/L	3.04	2.94	0.132	0.562
Low-density lipoprotein, mmol/L	1.18	1.23	0.090	0.731
Total bilirubin, μmol/L	2.71	2.81	0.185	0.710
Lactic acid, mmol/L	2.18^b^	2.64^a^	0.126	0.010
Alanine aminotransferase, U/L	24.8	24.5	0.63	0.688
Aspartate aminotransferase, U/L	72.1	75.9	2.43	0.257
Alkaline phosphatase, U/L	38.9	38.5	1.72	0.867
γ-glutamyl transpeptidase, U/L	30.4	32.4	1.63	0.368
Lactic dehydrogenase, U/L	1024.9	1037.5	22.30	0.680
Creatine kinase, U/L	164.4	166.0	8.60	0.892
Amylase, U/L	27.4	26.2	1.51	0.573
Hepatic lipase, U/L	3.29	3.37	0.130	0.620
Cholinesterase, U/L	126.9	132.8	3.70	0.250
Ca, mmol/L	2.12	2.14	0.034	0.624
P, mmol/L	1.81	1.80	0.039	0.785

a,b *Means within a row for treatments that do not have a common superscript differ*.

1 *SBM, soybean meal*;

2 *FSBM, fermented soybean meal*;

3 *SEM for treatments*.

### Summary of Metabolites Identified Across Four Biofluids

**Figure 1:**
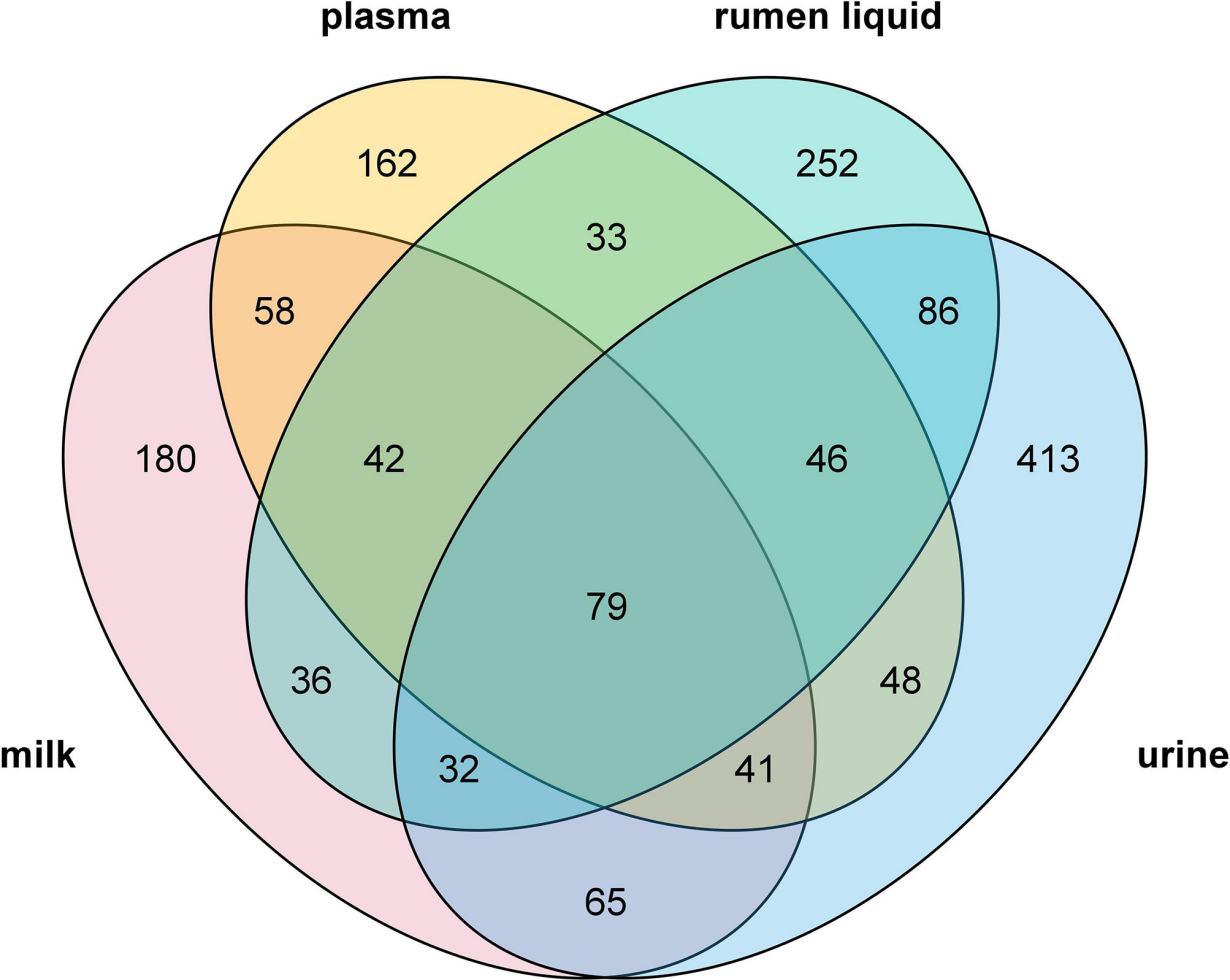
Venn diagram illustrating the unique and common metabolites detected in the rumen liquid, plasma, milk, and urine.

Overall, 913 metabolites were identified across the four biofluids (i.e, rumen liquid, plasma, milk, and urine) of dairy cows in this study, amongst which a total of 79 metabolites were shared by all the four biofluids, including benzoic acid, taurine, and L-phenylalanine (Figure 1). Besides, 252, 162, 180, and 413 metabolites were exclusive to rumen fluid, plasma, milk, and urine, respectively. The metabolites such as pregeijerene, anatabine, deoxyinosine, and other 249 metabolites generated during the rumen fermentation were only detected in rumen liquid, while stearoylcarnitine (2-Naphthalenyloxy)acetic acid, lutein, and 159 others were unique to plasma. Metabolites including hypogeic acid, 2-Methyl-4-oxopentanedioic acid, N-(3-Methylbutyl)acetamide and other 177 compounds produced during lactation were only identified in the milk, whereas 3-isoxazolidinone, mangostenol, kinetin, and 410 others were only discovered in urine. Furthermore, 42 compounds were common in rumen fluid, plasma, and milk, 46 metabolites were shared by rumen fluid, plasma, and urine, 32 compounds were common in rumen fluid, milk, and urine, whilst 41 metabolites were shared by plasma, milk, and urine, respectively. The numbers of the common compounds in each of the two biofluids are exhibited in Figure 1. The detailed information on all the detected metabolites across the four biofluids has been supplied in the Supplementary Table 2.

### Metabolic Pathways of Common Metabolites Across Four Biofluids

**Figure 2:**
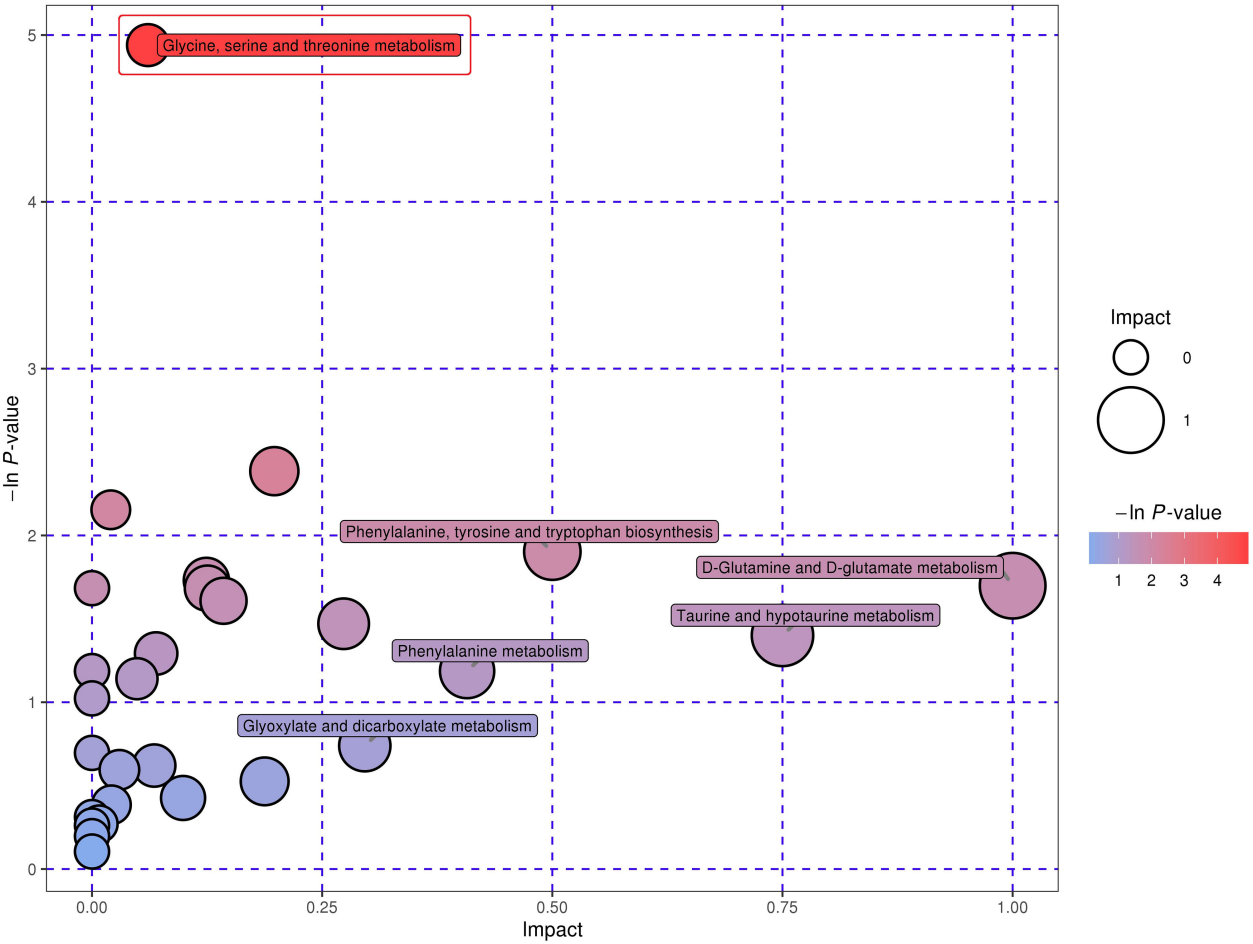
Bubble plot demonstrating the common metabolic pathways in the four biofluids across two dietary treatments. The x-axis symbolizes the pathway impact, whilst the y-axis represents the pathway enrichment. The bubbles with larger sizes and darker colors represent pathways with higher impact values and higher enrichment. The red frame indicates that the pathway inside was identified with an enrichment *p* < 0.05.

In total, 29 metabolic pathways for the 79 shared metabolites across all the four biofluids were identified, consisting of glycine, serine and threonine metabolism, arginine and proline metabolism, pantothenate and CoA biosynthesis, phenylalanine, tyrosine, and tryptophan biosynthesis, pyrimidine metabolism and other 24 metabolic pathways (Supplementary Table 3). Amongst those 29 pathways, D-glutamine and D-glutamate metabolism, taurine and hypotaurine metabolism, phenylalanine, tyrosine and tryptophan biosynthesis, phenylalanine metabolism, and glyoxylate and dicarboxylate metabolism were detected with the impact value of 1.00, 0.75, 0.50, 0.41, and 0.30, respectively. With the enrichment *p-*value of 0.007, the glycine, serine, and threonine metabolism could be defined as the most relevant pathway for the common metabolites in the four biofluids (Figure 2).

### Comparison of Metabolite Profiles Across Four Biofluids Between Treatments

**Figure 3:**
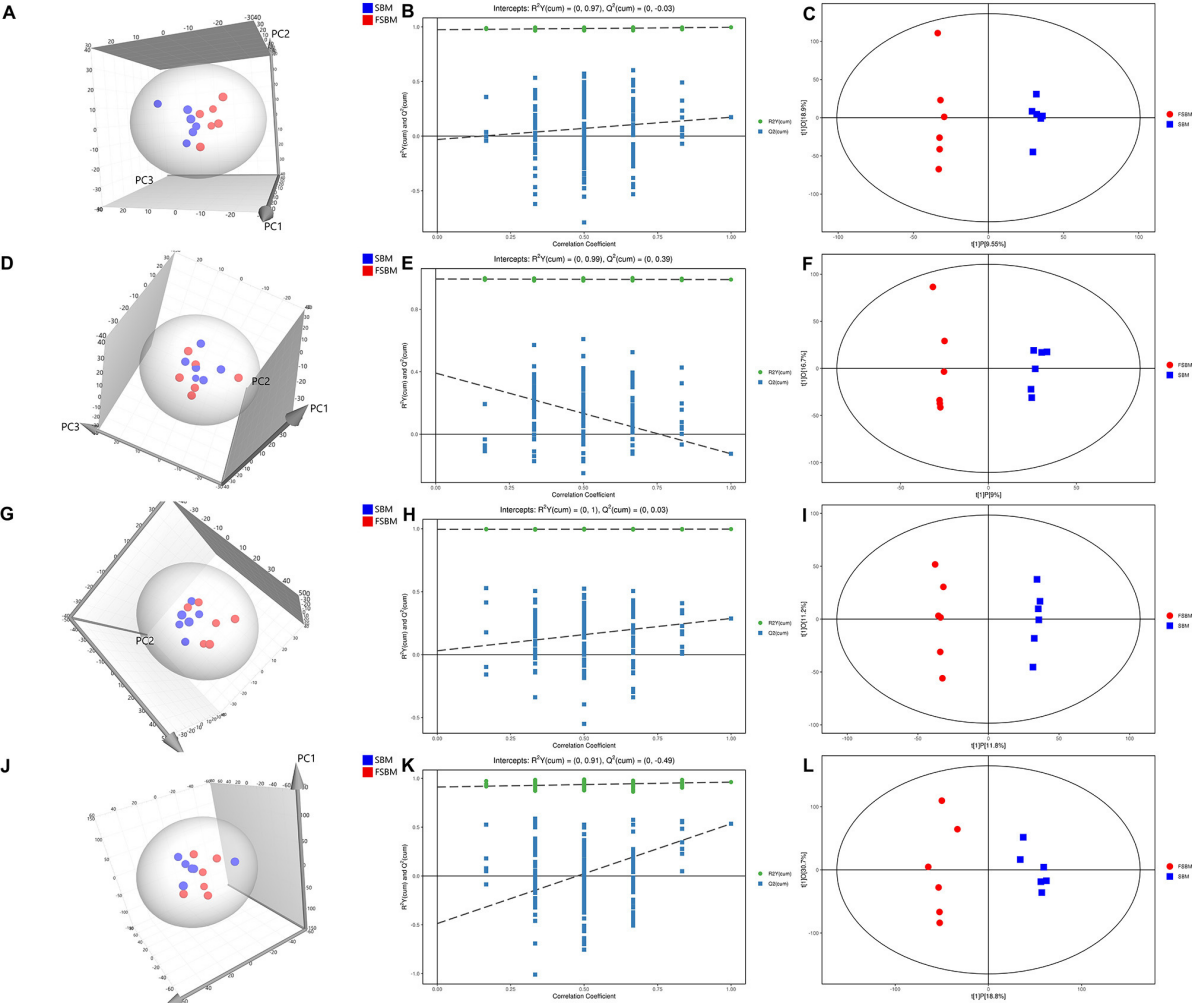
3D score scatter plots of principal component analysis (PCA) **(A,D,G,J)**, permutation test plots of orthogonal projections to latent structures-discriminant analysis (OPLS-DA) **(B,E,H,K)**, and OPLS-DA score scatter plots **(C,F,I,L)** for the metabolite profiles in rumen liquid **(A–C)**, plasma **(D–F)**, milk **(G–I)**, and urine **(J–L)** between two dietary treatments. SBM, soybean meal treatment; FSBM, fermented soybean meal treatment.

As depicted in Figures 3A,D,G,J, all the samples in the 3D score scatter PCA plots for the four biofluids were shown to be inside the 95% Hotelling's T-squared ellipse. It was noteworthy that the clustering of metabolite profiles from the two treatments was overlapped in plasma, milk, and urine, but a distinction for the samples in rumen fluid between the SBM and FSBM was also noticed. The models for the OPLS-DA analysis in differentiating the SBM and FSBM groups were examined through the permutation test (Figures 3B,E,H,K). The corresponding *R*^2^*Y* values for the OPLS-DA models of rumen liquid, plasma, milk, and urine were, respectively, 0.97, 0.99, 1.00, and 0.91, revealing the sufficient validity of the OPLS-DA models. The results of OPLS-DA analysis for rumen liquid, plasma, milk, and urine are shown in Figures 3C,F,I,L, respectively. All the samples from the four biofluids were within the 95% Hotelling's T-squared ellipse in the OPLS-DA score scatter plots, and a significant discrepancy in the metabolite profiles between the SBM and FSBM groups was noticeable in each biofluid.

### Differentially Expressed Metabolites in Four Biofluids Between Treatments

In sum, 40, 16, 43, and 111 significantly differential metabolites with the VIP value > 1 and the *p* < 0.05 were identified in rumen liquid, plasma, milk, and urine, respectively, between the FSBM and SBM treatments (Supplementary Table 4). More specifically, amongst the 40 significantly different metabolites in the rumen liquid, 28 metabolites were discovered with a higher abundance in the FSBM-fed dairy cows than that in the SBM dairy cows. The majority of those 28 metabolites were classified as amino acids, peptides, and analogs, glycerophosphoethanolamines, or fatty alcohols. In the plasma, the amounts of the 8 molecules among the 16 differentially expressed metabolites were higher in the FSBM treatment compared with the SBM group, with 2 significant compounds belonging to the eicosanoids at the Sub Class level. As to the 43 significantly different metabolites in the milk, 26 compounds were detected at higher levels in the FSBM-fed cows than in the SBM cows, and most of them were assigned as carbohydrates and carbohydrate conjugates, fatty acid esters, fatty acids and conjugates, glycerophosphoethanolamines, or glycerophosphoserines. Out of the 111 significantly differential molecules in the urine, only 28 metabolites primarily comprised of carbohydrates and carbohydrate conjugates, amino acids, peptides, and analogs, or purine nucleosides were identified with higher concentrations in the FSBM group than in the SBM group. As for those 83 metabolites detected with significantly less amounts in the urine of the FSBM-fed cattle than the SBM-fed cattle, the top four categories at the Sub Class level were successively amino acids, peptides, and analogs, fatty acids and conjugates, carbohydrates and carbohydrate conjugates, and carbonyl compounds.

### Differential Metabolic Pathways in Four Biofluids Between Treatments

**Figure 4:**
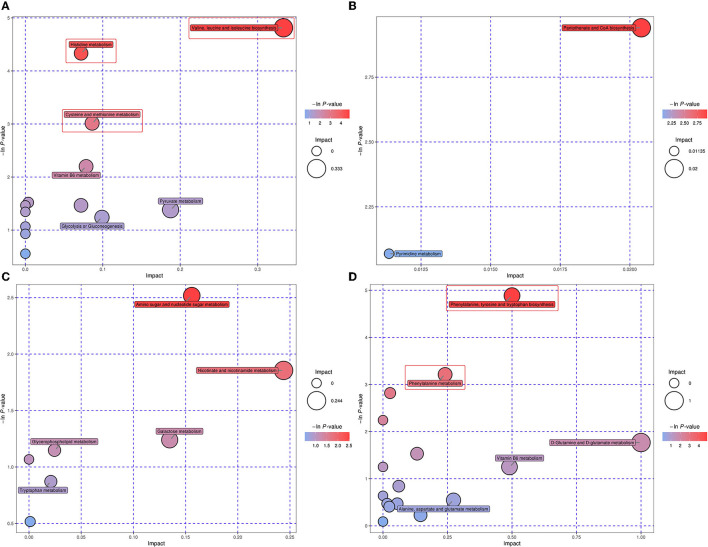
Bubble plot demonstrating the differential metabolic pathways between the two dietary treatments in rumen liquid **(A)**, plasma **(B)**, milk **(C)**, and urine **(D)**. The x-axis symbolizes the pathway impact, whilst the y-axis represents the pathway enrichment. The bubbles with larger sizes and darker colors represent pathways with higher impact values and higher enrichment. The red frame indicates that the pathway inside was identified with an enrichment *p* < 0.05.

As revealed through the KEGG pathway annotation and the subsequent metabolic pathway analysis, 13, 2, 7, and 16 differential metabolic pathways were identified according to the significantly differential metabolites in the rumen fluid, plasma, milk, and urine, respectively (Supplementary Table 5). In the rumen liquid, the valine, leucine, and isoleucine biosynthesis, histidine metabolism, and cysteine and methionine metabolism were the three most relevant differential pathways with the enrichment *p* < 0.05 (Figure 4A). For the differential pathways in plasma, both the pantothenate and CoA biosynthesis and pyrimidine metabolism were detected with an enrichment *p* > 0.05 (Figure 4B). Similarly, in the milk, none of the seven differential pathways was identified with the corresponding enrichment *p* < 0.05 (Figure 4C). Nevertheless, the metabolic pathways of phenylalanine, tyrosine and tryptophan biosynthesis, and phenylalanine metabolism were the two most significantly different pathways in the urine, with the enrichment *p-*values at 0.008 and 0.040, respectively (Figure 4D).

Furthermore, based on the significantly different metabolites and the metabolic pathway analysis, a total of 10 common differential metabolic pathways across different biofluids were identified (Table 4). The alanine, aspartate, and glutamate metabolism, aminoacyl-tRNA biosynthesis, butanoate metabolism, and vitamin B6 metabolism were detected as the common different pathways across the rumen fluid and urine. Whilst in both rumen liquid and milk, glycine, serine, and threonine metabolism, and purine metabolism were the two mutually differential pathways. The pantothenate and CoA biosynthesis was identified as the unique different pathway shared by the plasma and urine. In addition, galactose metabolism, glycerophospholipid metabolism, and tryptophan metabolism were the common differential pathways within the biofluids of milk and urine.

**Table 4:** Common differential metabolic pathways identified from the significantly different metabolites across different biofluids between the SBM group and FSBM group.

**Metabolic pathway**	**Biofluid**	**Metabolite**
Alanine, aspartate and glutamate metabolism	Rumen liquid	Pyruvic acid (1.736)^a^
	Urine	L-Glutamic acid (0.505)
Aminoacyl-tRNA biosynthesis	Rumen liquid	L-Isoleucine (1.228)
	Urine	L-Tyrosine (0.471)
Butanoate metabolism	Rumen liquid	Pyruvic acid (1.736)
	Urine	L-Glutamic acid (0.505)
Vitamin B6 metabolism	Rumen liquid	Pyridoxamine (1.245)
	Urine	Pyridoxal (0.708)
Glycine, serine and threonine metabolism	Rumen liquid	Pyruvic acid (1.736)
	Milk	Choline (0.902)
Purine metabolism	Rumen liquid	Adenosine (0.444)
		Inosine (0.489)
	Milk	Adenosine (1.397)
Pantothenate and CoA biosynthesis	Plasma	Pantothenic acid (1.261)
	Urine	D-4'-Phosphopantothenate (0.348)
Galactose metabolism	Milk	Galactose 1-phosphate (7.539)
	Urine	Alpha-Lactose (1.409)
Glycerophospholipid metabolism	Milk	Choline (0.902)
	Urine	Glycerylphosphorylethanolamine (1.376)
Tryptophan metabolism	Milk	Indoleacetaldehyde (0.516)
	Urine	5-Hydroxyindoleacetic acid (0.545)
		3-Hydroxyanthranilic acid (0.726)
		Indoleacetic acid (0.765)
		N-Methyltryptamine (0.594)

a *The value in the parentheses is the fold change of the corresponding metabolite, which is the ratio of the quantity of the metabolite in the FSBM group to that in the SBM group. It means that the amount of the metabolite is higher in the FSBM treatment than that in the SBM treatment, when the corresponding FC value is above 1*.

### Integrated Key Differential Pathways Between Treatments

**Figure 5:**
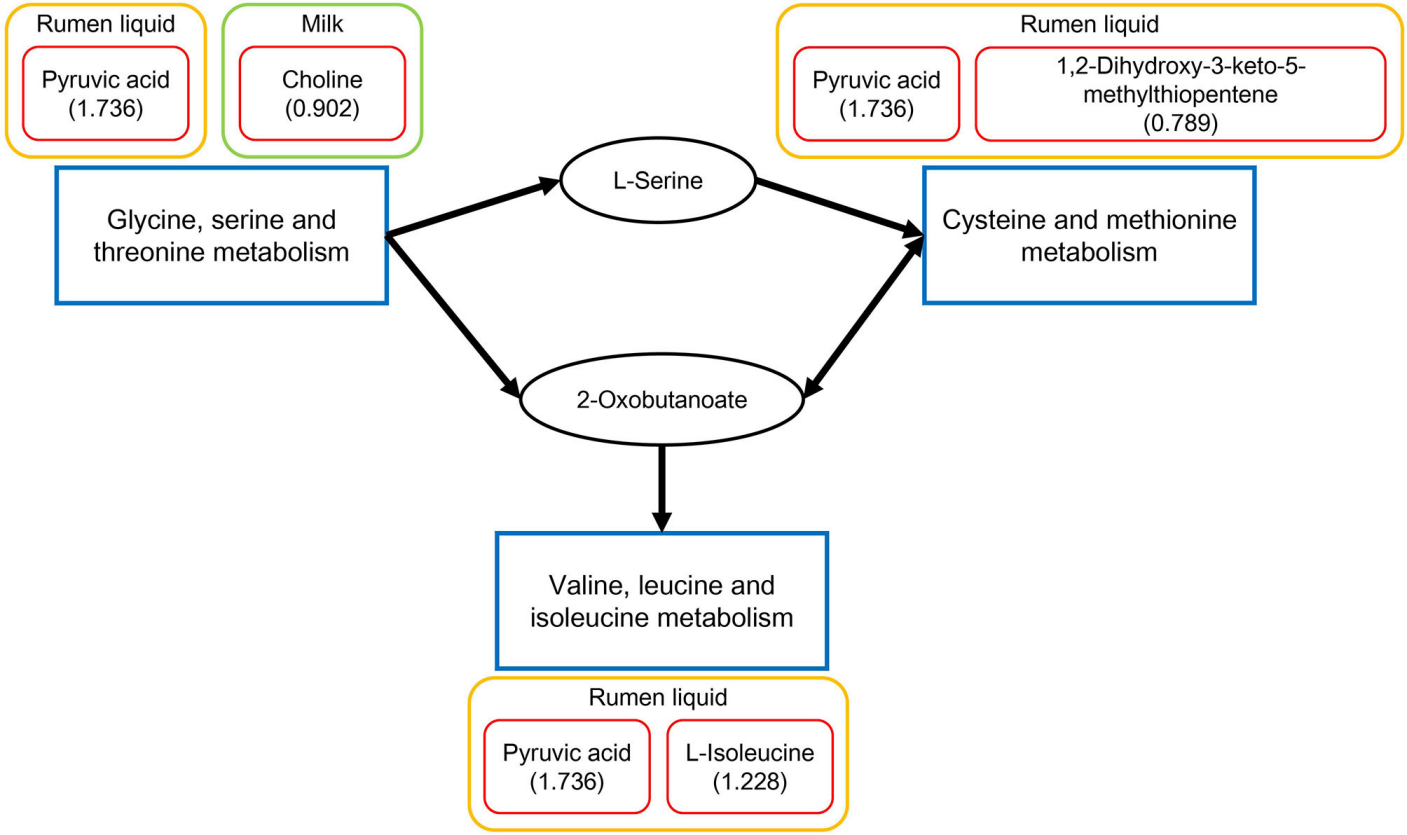
Integrated key differential metabolic pathways and relevant significantly different metabolites in each biofluid between the two dietary treatments. The significantly different metabolites in each biofluid for each pathway are displayed in the red boxes. Each number in the parentheses is the fold change of the corresponding metabolite, which is the ratio of the quantity of the metabolite in the FSBM group to that in the SBM group.

According to the above-mentioned results of the relevant pathways from the common metabolites in the four biofluids, significantly different pathways in each biofluid, and commonly differential metabolic pathways across different biofluids, the critical differential metabolic pathways were integrated by referring to the KEGG database. As depicted in Figure 5, the glycine, serine, and threonine metabolism, valine, leucine, and isoleucine biosynthesis, and cysteine and methionine metabolism were the three key differential pathways that could be connected together based on the reference map of the KEGG database, with the involved differentially expressed metabolites presented alongside.

## Discussion

Because of the lower concentration of anti-nutritional agents, higher level of RUP, and more amounts of non-protein nitrogen (e.g., small peptides, free amino acids, and ammonia) and vitamins, the FSBM is hence considered as a more favored protein feed for domestic ruminants when compared with the SBM (6, 10, 11). However, the overwhelming majority of previous studies on the replacement of SBM by FSBM were conducted in young calves, mainly aiming at its effects on the growth performance and immune-physiological responses (3, 12, 13, 35). By contrast, the reactions of the mature ruminants including lactating cattle to the dietary substitution of FSBM for SBM have been seldom investigated. In this experiment, we first observed that the FSBM replacement reduced the dry matter intake of dairy cows without altering the 4% fat-corrected milk yield, milk efficiency, and nutrients digestibility (data submitted elsewhere). For the FA profiles in the milk, it was observed that substituting FSBM for SBM in the diet for lactating Holstein dairy cows significantly raised or tended to increase the concentrations of C13:0, C14:0, C14:1, and C16:0, which could be roughly assigned to the medium-chain FA (36). As reported previously, the above up-regulated medium-chain FA are principally produced through the *de novo* synthesis from the substrates such as acetate and butyrate within the mammary gland (37, 38). Therefore, it could be inferred that the absorption of acetate and/or butyrate via the rumen epithelium and the subsequent utilization was enhanced when the SBM was entirely replaced by FSBM in the current study. This assumption could be a possible explanation for the lower proportion of butyrate present in the rumen fluid of the FSBM-fed lactating cows, as revealed in our precedent report (14). Furthermore, we had previously found that replacing SBM with FSBM in the diet for lactating dairy cows promoted either the copy numbers or abundances of *Fibrobacter succinogenes, Selenomonas ruminantium, Prevotella* spp., and *Saccharofermentans acetigenes* in the rumen liquid (14), all of which have been identified as the predominant acetate-producers within the ruminal microflora (37, 39, 40). The enrichment of those ruminal bacteria could probably provide the host with more acetate as the precursor for the *de novo* synthesis of the medium-chain FA in the mammary gland. In addition, the declines in the ratios of a few long-chain FA (i.e., C17:0, C18:0, C18:1n9c, and C20:0) of this trial might probably imply a reduction of the mobilization from triglycerides in the adipose tissue (38), further indicating a possible positive energy balance during galactosis of the host (28). However, the verification of this assumption requires further investigations.

As reported previously, the regular range of blood urea nitrogen for dairy cows is ~2.14–9.64 mmol/L (41). In the current study, the levels of blood urea nitrogen in the two dietary treatments both fell within this physiological range. Besides, the significant increment of blood urea nitrogen in the FSBM-fed cows was likely attributed to the greater amounts of non-protein nitrogen in the FSBM than SBM and promoted translocation of ruminal nitrogen into the blood in the dairy cows from the FSBM group compared to the SBM group (41, 42). Kulka et al. (43) suggested that uric acid in the blood could be taken as an indicator of the anti-oxidative status of the dairy cow, since monoanion urate, the dissociated form of uric acid, plays a significant role in the anti-oxidative protection during lactation when dynamic metabolic alterations occur. It was noticed in this trial that replacing SBM with FSBM tended to increase the quantity of blood uric acid, which might be correlated to the enhancement of anti-oxidative protection of the FSBM-fed cattle but necessitates further explorations. Moreover, the concentration of lactic acid in the blood of dairy cattle rose in response to the replacement of SBM with FSBM in this study. A possible cause for this phenomenon could be the fact that the FSBM used in this trial was fermented with the inoculation of *Lactobacillus* spp., which certainly enhanced the amount of lactic acid in FSBM compared with SBM. Besides, the surges in the copy numbers or abundances of *Selenomonas ruminantium* and *Saccharofermentans acetigenes* in the rumen fluid of FSBM-fed dairy cows in our previous report (14) might also account for the higher levels of blood lactic acid, since these two ruminal bacterial species are characterized by the capacity of yielding lactate during fermentation (37, 39). Rezazadeh et al. (13) found that the substitution of FSBM for SBM at 50% in the starter diet reduced the density of serum glucose in the abruptly weaned Holstein calves under cold conditions, which was similar to the finding of the present study that the amount of glucose in the blood of dairy cows from the FSBM treatment was less than the SBM treatment. This reaction may secure the availability of ruminal fermentation outputs as the fundamental energy source (44), and also result from the relieving stress of the FSBM-fed cattle. It was worth mentioning that a higher ruminal propionate concentration in the FSBM-fed cows than the SBM-fed counterparts was marked in our precedent research (14), which could probably represent fewer propionate absorbed through the ruminal epithelium, and thence reduced hepatic gluconeogenesis with less propionate as the substrate (37, 45). However, the above hypotheses require further investigations to be verified.

In the past few years, metabolomics analysis has been introduced in the studies on the metabolisms of dairy cattle under varying conditions (46, 47). As an approach simultaneously targeting the metabolism of different biofluids, the multiple biofluid metabolomics could offer a more comprehensive insight into the internal metabolic status of dairy cows (17, 18, 22). This present study, to our knowledge, is the first attempt in investigating the deep impacts of replacing SBM with FSBM on the metabolism of lactating dairy cattle through a multiple biofluid metabolomics analysis simultaneously aimed at the rumen liquid, plasma, milk, and urine. Sun et al. (17) concluded that the gas chromatography time-of-flight/mass spectrometry (GC-TOF/MS) based metabolomics is more capable of detecting low-concentration and small-molecular metabolites and therefore it can identify more metabolites, when compared with the metabolomics performed on other platforms such as the nuclear magnetic resonance (NMR), inductively coupled plasma mass spectroscopy (ICP-MS), and gas chromatography-mass spectrometry (GC-MS) (47–49). In this experiment, with the aid of UHPLC-MS-based metabolomics approach, a total of 606, 509, 533, and 810 metabolites were identified in the rumen liquid, plasma, milk, and urine, respectively. The numbers of the compounds detected in each biofluid were much greater than those reported in the previous investigations, indicating the improved detection and quantification of the metabolites across biofluids (17, 18, 50–52). Besides, this investigation identified 79 compounds that co-existed across the four different biofluids and consequently detected 29 corresponding metabolic pathways, amongst which glycine, serine, and threonine metabolism were considered the most relevant pathway for the mutual metabolites in the four biofluids of the dairy cows. This finding confirmed the correlations among the metabolisms within the four biofluids and requires further explorations.

As was illustrated in the OPLS-DA plots of the current trial, the clustering of the samples from the FSBM group was distinct from that from the SBM group in each of the biofluids, suggesting that the substitution of FSBM for SBM altered the metabolic profiles of all the four biofluids in dairy cattle. Furthermore, 40, 16, 43, and 111 significantly differential metabolites were discovered in the rumen fluid, plasma, milk, and urine, respectively. In the rumen liquid, it was noteworthy that amongst the 28 identified with higher concentrations in the FSBM treatment than the SBM group, six differential compounds that took up the largest proportion were classified as amino acids, peptides, and analogs. This result was in line with the greater amounts of small peptides and free amino acids in FSBM compared with SBM, implying that more amino acids and peptides would be provided as substrates for the microbial protein synthesis of the ruminal microorganism, and thence more microbial protein produced for the host (53). Within those eight metabolites whose densities were higher in the plasma of FSBM-fed cows than the SBM-fed ones, two compounds (i.e., 8-isoprostaglandin E1 and 19-hydroxy-PGE2) were assigned to the category of eicosanoids. As a potent proinflammatory mediator, eicosanoid is believed to possess the capacity of either augmenting or alleviating inflammatory responses of dairy cattle (54). The effects of these two eicosanoids that increased in the blood of the FSBM-fed cattle were uncertain, necessitating future studies to be uncovered.

The metabolic profile of milk could be affected by a variety of factors (e.g., breeds, diets, lactation stages, and environments), and it is tightly bound up with the quality of the milk secreted by the dairy cattle (18, 46, 51). In the current study, the 26 metabolites which significantly raised in the milk of cows in response to the FSBM replacement were predominantly comprised of carbohydrates and carbohydrate conjugates, fatty acid esters, and fatty acids and conjugates. The growth in the abundances of these molecules may be related to the aforementioned increases of the medium-chain FA in the FSBM-fed cows, but it requires further research to be examined. Due to its advantages in sampling, pretreatment, and preservation, urine metabolomics has been widely adopted in detecting biomarkers and interpreting the physiological activities of animals under varied conditions (18, 55). In this trial, it was observed that only 28 of the 111 significantly differential metabolites in the urine were increased by substituting FSBM for SBM, which was opposite to the variations in the other three biofluids. Furthermore, the 19 compounds belonging to the amino acids, peptides, and analogs occupied the greatest portion out of the 83 metabolites identified with fewer amounts in the urine of the cows fed FSBM. This phenomenon might indicate an improved nitrogen utilization of the dairy cattle since less amino acids and peptides were excreted through the urination (55). It should be taken into account that, attributed to the substantial increment in the number of different metabolites identified across the four biofluids with a more sensitive metabolomics platform compared with previous reports, the level of the difficulties in defining and understanding the differential molecules as biomarkers in response to the dietary change also rose dramatically. Therefore, more deep investigations are required to disclose the exact roles and relevant mechanisms of those biomarkers during the physiological process in dairy cows.

In this experiment, 3 and 2 significantly different metabolic pathways were identified in the rumen fluid and urine, respectively. Further, all those differential pathways were related to amino acid metabolism. More specifically, for the significantly differential pathway of valine, leucine, and isoleucine biosynthesis in rumen liquid, the different metabolites L-isoleucine [fold change (FC) = 1.228] and pyruvic acid (FC = 2.024) were involved. Precedent studies have confirmed that pyruvate, the conjugated base of pyruvic acid, is the main precursor for the production of valine (52). The increases in the concentrations of L-isoleucine and pyruvic acid implied that the biosynthesis of the essential amino acids valine, leucine, and isoleucine in the rumen of dairy cows could be due to some extent promoted by the FSBM replacement. Besides, the imidazole-4-acetaldehyde (FC = 0.788) and formiminoglutamic acid (FC = 0.649) were related to the histidine metabolism significantly influenced by substituting FSBM for SBM. Being an essential amino acid, histidine is not only the precursor of intermediates of the tricarboxylic acid cycle but also a substrate for histamine and carnosine biosynthesis as an inflammatory agent (20). Moreover, the ruminal cysteine and methionine metabolism was also significantly altered by the FSBM replacement, according to the corresponding differential compounds of 1,2-dihydroxy-3-keto-5-methylthiopentene (FC = 0.789) and pyruvic acid (FC = 2.024). Both cysteine and methionine are indispensable for the protein production of immune functions, and the latter can be converted to the former via the trans-sulfide pathway (56). Methionine plays a vital role in the inflammatory reactions, as the precursor of acetylcholine and phosphatidylcholine. While cysteine is considered a significant antioxidative agent since it is the substrate for glutathione synthesis (56). Considering the result that the ruminal metabolisms of histidine, cysteine and methionine were altered, it could be speculated that replacing SBM with FSBM might possibly exert influences on the inflammatory responses and antioxidative status of the dairy cattle in the present study.

In this trial, the urinary phenylalanine, tyrosine, and tryptophan biosynthesis, and phenylalanine metabolism were the two significantly different pathways, both with the involvement of different metabolites phenylpyruvic acid (FC = 0.517) and L-tyrosine (FC = 0.471). The concentrations of these two molecules in the urine of FSBM-fed cows were both lower than those in the SBM-fed ones. Phenylalanine, tyrosine, and tryptophan are aromatic amino acids, all essential for protein synthesis. More specifically, phenylalanine is the substrate for the generation of catecholamine, a vital neurotransmitter (57). Through hydroxylation, phenylalanine can be converted to tyrosine, which is the precursor of hormones including catechol estrogens and thyroid (58). Tryptophan can be used to produce a few bioactive compounds which participate in various physiological processes, such as immune responses, inflammation, neurotransmission, and growth regulation (59). The less amounts of urinary L-tyrosine and phenylpyruvic acid existing could result from the enhanced utilization efficiency of phenylalanine, tyrosine, and tryptophan in the FSBM-fed cows' liver, where the degradation and utilization of most of the amino acids take place (17).

In the current study, according to the findings on the relevant pathways from the common metabolites in the four biofluids, significantly different pathways in each biofluid, and commonly differential metabolic pathways across different biofluids, we assembled three key different pathways across biofluids: the glycine, serine, and threonine metabolism, valine, leucine and isoleucine biosynthesis, and cysteine and methionine metabolism. Amongst them, glycine, serine, and threonine metabolism was the significantly relevant pathway for the overlapping metabolites in the four biofluids, as well as a differential pathway identified in both rumen fluid and milk. In a previous investigation, Sun et al. (17) identified the glycine, serine, and threonine metabolism as the significantly relevant pathway of common metabolites in rumen fluid, milk, serum, and urine of Holstein dairy cows, and also a key different pathway across all the four biofluids between the cattle fed alfalfa hay and cattle fed corn stover. Based on the connections within the aforementioned three integrated key differential pathways, we assume that those three pathways may play an important role in the metabolic responses of dairy cows to the substitution of FSBM for SBM. However, the detailed alterations of these pathways and their correlations in response to the FSBM replacement need further studies to be investigated.

## Conclusion

Replacing SBM with FSBM raised the concentrations of a few medium-chain FA (i.e., C13:0, C14:0, C14:1, and C16:0), but reduced the levels of several long-chain FA (i.e., C17:0, C18:0, C18:1n9c, and C20:0) in the milk. Besides, the increments in the amounts of urea nitrogen lactic acid, and uric acid, and the decline in the density of glucose in the blood in response to the FSBM replacement were also noticed. It was further revealed that substituting FSBM for SBM altered the metabolic profiles of all the four biofluids. Based on the identified differentially expressed metabolites, we, respectively, detected 3 and 2 significantly different metabolic pathways between the FSBM and SBM treatments in the rumen fluid and urine, which were all related to the metabolism of amino acids. Moreover, glycine, serine, and threonine metabolism, valine, leucine, and isoleucine biosynthesis, and cysteine and methionine metabolism were the three key integrated different pathways in this trial. The present study mainly indicated that the FSBM replacement could improve the nitrogen utilization efficiency, and probably influence the inflammatory responses and antioxidative functions of dairy cows. Further research works are necessitated to uncover the exact effects and correlations of the differential metabolites and pathways detected in this trial.

## Data Availability Statement

The original contributions presented in the study are included in the article/Supplementary Material, further inquiries can be directed to the corresponding author/s.

## Ethics Statement

The animal study was reviewed and approved by Animal Care Committee, College of Animal Science and Technology, Hunan Agricultural University.

## Author Contributions

ZW, ZT, ST, JH, and FW designed the research. ZW, YY, WS, ST, and HY conducted the research. ZW, YY, and ST analyzed the data. ZW and ST wrote the paper. All authors approved the final manuscript.

## Funding

This work received funding through the Hunan Provincial Education Department (Grant No. 19B257), Hunan Provincial Natural Science Foundation (Grant Nos. 2019JJ50279 and 2019RS3021), Hunan Provincial Science and Technology Department (Grant No. 2017NK1020), and National Natural Science Foundation of China (Grant No. 31772633).

## Conflict of Interest

HY was a member of the Nanshan Dairy Company. The remaining authors declare that the research was conducted in the absence of any commercial or financial relationships that could be construed as a potential conflict of interest.

## Publisher's Note

All claims expressed in this article are solely those of the authors and do not necessarily represent those of their affiliated organizations, or those of the publisher, the editors and the reviewers. Any product that may be evaluated in this article, or claim that may be made by its manufacturer, is not guaranteed or endorsed by the publisher.

## References

[1] Imran M Shahid MQ Pasha TN Haque MN. Effects of replacing soybean meal with corn gluten meal on milk production and nitrogen efficiency in Holstein cows. SA J An Sci. (2018) 48:590. 10.4314/sajas.v48i3.20

[2] Liu YG Peng HH Schwab CG. Enhancing the productivity of dairy cows using amino acids. Anim Prod Sci. (2013) 53:1156–9. 10.1071/AN13203

[3] Kim MH Yun CH Lee CH Ha JK. The effects of fermented soybean meal on immunophysiological and stress-related parameters in Holstein calves after weaning. J Dairy Sci. (2012) 95:5203–12. 10.3168/jds.2012-5317 22916926

[4] Rego OA Regalo SMM Rosa HJD Alves SP Borba AES Bessa RJB. Effects of grass silage and soybean meal supplementation on milk production and milk fatty acid profiles of grazing dairy cows. J Dairy Sci. (2008) 91:2736–43. 10.3168/jds.2007-0786 18565932

[5] Yoo JS Jang HD Cho JH Lee JH Kim IH. Effects of fermented soy protein on nitrogen balance and apparent fecal and ileal digestibility in weaned pigs. Asian Australas J Anim Sci. (2009) 22:1167–73. 10.5713/ajas.2009.80274

[6] Zhang HY Yi JQ Piao XS Li PF Zeng ZK Wang D. The metabolizable energy value, standardized ileal digestibility of amino acids in soybean meal, soy protein concentrate and fermented soybean meal, and the application of these products in early-weaned piglets. Asian Australas J Anim Sci. (2013) 26:691–9. 10.5713/ajas.2012.12429 25049840 PMC4093336

[7] Chatterjee C Gleddie S Xiao C-W. Soybean bioactive peptides and their functional properties. Nutrients. (2018) 10:1211. 10.3390/nu10091211 30200502 PMC6164536

[8] Stein HH Berger LL Drackley JK Fahey GC Hernot DC Parsons CM. Nutritional properties and feeding values of soybeans and their coproducts. In: Johnson LA White PJ Gallowat R, editors. Soybeans. Amsterdam: Elsevier (2008). p. 613–60.

[9] Feng J Liu X Xu ZR Lu YP Liu YY. Effect of fermented soybean meal on intestinal morphology and digestive enzyme activities in weaned piglets. Dig Dis Sci. (2007) 52:1845–50. 10.1007/s10620-006-9705-0 17410452

[10] Wang W Wang Y Hao X Duan Y Meng Z An X. Dietary fermented soybean meal replacement alleviates diarrhea in weaned piglets challenged with enterotoxigenic Escherichia coli K88 by modulating inflammatory cytokine levels and cecal microbiota composition. BMC Vet Res. (2020) 16:245. 10.1186/s12917-020-02466-5 32664940 PMC7362456

[11] Feizi LK Zad SS Jalali SAH Rafiee H Jazi MB Sadeghi K. Fermented soybean meal affects the ruminal fermentation and the abundance of selected bacterial species in Holstein calves: a multilevel analysis. Sci Rep. (2020) 10:12062. 10.1038/s41598-020-68778-6 32694544 PMC7374609

[12] Kwon IH Kim MH Yun C-H Go JY Lee CH Lee HJ. Effects of fermented soybean meal on immune response of weaned calves with experimentally induced lipopolysaccharide challenge. Asian Australas J Anim Sci. (2011) 24:957–64. 10.5713/ajas.2011.10419

[13] Rezazadeh F Kowsar R Rafiee H Riasi A. Fermentation of soybean meal improves growth performance and immune response of abruptly weaned Holstein calves during cold weather. Anim Feed Sci Technol. (2019) 254:114206. 10.1016/j.anifeedsci.2019.114206

[14] Wang Z Yu Y Li X Xiao H Zhang P Shen W. Fermented soybean meal replacement in the diet of lactating holstein dairy cows: modulated rumen fermentation and ruminal microflora. Front Microbiol. (2021) 12:625857. 10.3389/fmicb.2021.625857 33584627 PMC7879537

[15] Benchaar C Calsamiglia S Chaves AV Fraser GR Colombatto D McAllister TA. A review of plant-derived essential oils in ruminant nutrition and production. Anim Feed Sci Technol. (2008) 145:209–28. 10.1016/j.anifeedsci.2007.04.01422444918

[16] Johnson CH Ivanisevic J Siuzdak G. Metabolomics: beyond biomarkers and towards mechanisms. Nat Rev Mol Cell Biol. (2016) 17:451–9. 10.1038/nrm.2016.25 26979502 PMC5729912

[17] Sun H-Z Wang D-M Wang B Wang J-K Liu H-Y Guan LL. Metabolomics of four biofluids from dairy cows: potential biomarkers for milk production and quality. J Proteome Res. (2015) 14:1287–98. 10.1021/pr501305g 25599412

[18] Kim HS Kim ET Eom JS Choi YY Lee SJ Lee SS. Exploration of metabolite profiles in the biofluids of dairy cows by proton nuclear magnetic resonance analysis. PLoS ONE. (2021) 16:e0246290. 10.1371/journal.pone.0246290 33513207 PMC7845951

[19] Sun H Wang B Wang J Liu H Liu J. Biomarker and pathway analyses of urine metabolomics in dairy cows when corn stover replaces alfalfa hay. J Anim Sci Biotechnol. (2016) 7:49. 10.1186/s40104-016-0107-7 27583137 PMC5006375

[20] Sun H-Z Shi K Wu X-H Xue M-Y Wei Z-H Liu J-X. Lactation-related metabolic mechanism investigated based on mammary gland metabolomics and 4 biofluids' metabolomics relationships in dairy cows. BMC Genomics. (2017) 18:936. 10.1186/s12864-017-4314-1 29197344 PMC5712200

[21] Wang H He Y Li H Wu F Qiu Q Niu W. Rumen fermentation, intramuscular fat fatty acid profiles and related rumen bacterial populations of Holstein bulls fed diets with different energy levels. Appl Microbiol Biotechnol. (2019) 103:4931–42. 10.1007/s00253-019-09839-3 31020378

[22] Yue S Ding S Zhou J Yang C Hu X Zhao X. Metabolomics approach explore diagnostic biomarkers and metabolic changes in heat-stressed dairy cows. Animals. (2020) 10:1741. 10.3390/ani10101741 32992834 PMC7601318

[23] Zhang G Mandal R Wishart DS Ametaj BN. A multi-platform metabolomics approach identifies urinary metabolite signatures that differentiate ketotic from healthy dairy cows. Front Vet Sci. (2021) 8:595983. 10.3389/fvets.2021.595983 33575283 PMC7871000

[24] Shen JS Chai Z Song LJ Liu JX Wu YM. Insertion depth of oral stomach tubes may affect the fermentation parameters of ruminal fluid collected in dairy cows. J Dairy Sci. (2012) 95:5978–84. 10.3168/jds.2012-5499 22921624

[25] AOAC. Official Methods of Analysis. Gaithersburg, MD: AOAC Int. (2005).

[26] Tang SX He Y Zhang PH Jiao JZ Han XF Yan QX. Nutrient digestion, rumen fermentation and performance as ramie (*Boehmeria nivea*) is increased in the diets of goats. Anim Feed Sci Technol. (2019) 247:15–22. 10.1016/j.anifeedsci.2018.10.013

[27] Wang Z Li XY Yu YN Yang LY Zhang PH He JH. Enhancing dietary cation-anion difference reshaped the distribution of endotoxin across different biofluids and influenced inflammatory response in dairy cows exposed to heat stress. Anim Feed Sci Technol. (2020) 262:114444. 10.1016/j.anifeedsci.2020.114444

[28] Billa PA Faulconnier Y Larsen T Leroux C Pires JAA. Milk metabolites as noninvasive indicators of nutritional status of mid-lactation Holstein and Montbéliarde cows. J Dairy Sci. (2020) 103:3133–46. 10.3168/jds.2019-17466 32059860

[29] Dewanckele L Vlaeminck B Hernandez-Sanabria E Ruiz-González A Debruyne S Jeyanathan J. Rumen biohydrogenation and microbial community changes upon early life supplementation of 22:6n-3 enriched microalgae to goats. Front Microbiol. (2018) 9:573. 10.3389/fmicb.2018.00573 29636742 PMC5880937

[30] Lerch S Ferlay A Shingfield KJ Martin B Pomiès D Chilliard Y. Rapeseed or linseed supplements in grass-based diets: effects on milk fatty acid composition of Holstein cows over two consecutive lactations. J Dairy Sci. (2012) 95:5221–241. 10.3168/jds.2012-5337 22916928

[31] Guzmán JL Perez-Ecija A Zarazaga LA Martín-García AI Horcada A Delgado-Pertíñez M. Using dried orange pulp in the diet of dairy goats: effects on milk yield and composition and blood parameters of dams and growth performance and carcass quality of kids. Animal. (2020) 14:2212–20. 10.1017/S1751731120000932 32367792

[32] Lin F Cai F Luo B Gu R Ahmed S Long C. Variation of microbiological and biochemical profiles of laowo dry-cured ham, an indigenous fermented food, during ripening by GC-TOF-MS and UPLC-QTOF-MS. J Agric Food Chem. (2020) 68:8925–35. 10.1021/acs.jafc.0c03254 32706588

[33] Xia J Sinelnikov IV Han B Wishart DS. MetaboAnalyst 3.0—making metabolomics more meaningful. Nucleic Acids Res. (2015) 43:W251–7. 10.1093/nar/gkv380 25897128 PMC4489235

[34] Kanehisa M Sato Y Kawashima M Furumichi M Tanabe M. KEGG as a reference resource for gene and protein annotation. Nucleic Acids Res. (2016) 44:D457–62. 10.1093/nar/gkv1070 26476454 PMC4702792

[35] Kim MH Yun CH Kim HS Kim JH Kang SJ Lee CH. Effects of fermented soybean meal on growth performance, diarrheal incidence and immune-response of neonatal calves: effect of FSBM on immune response in calves. Anim Sci J. (2010) 81:475–81. 10.1111/j.1740-0929.2010.00760.x 20662817

[36] Glasser F Ferlay A Doreau M Schmidely P Sauvant D Chilliard Y. Long-chain fatty acid metabolism in dairy cows: a meta-analysis of milk fatty acid yield in relation to duodenal flows and *de novo* synthesis. J Dairy Sci. (2008) 91:2771–85. 10.3168/jds.2007-0383 18565935

[37] Fievez V Colman E Castro-Montoya JM Stefanov I Vlaeminck B. Milk odd- and branched-chain fatty acids as biomarkers of rumen function—an update. Anim Feed Sci Technol. (2012) 172:51–65. 10.1016/j.anifeedsci.2011.12.008

[38] Vyas D Teter BB Erdman RA. Milk fat responses to dietary supplementation of short- and medium-chain fatty acids in lactating dairy cows. J Dairy Sci. (2012) 95:5194–202. 10.3168/jds.2011-5277 22916925

[39] Chen S Niu L Zhang Y. *Saccharofermentans acetigenes* gen. nov., sp. nov., an anaerobic bacterium isolated from sludge treating brewery wastewater. Int J Syst Evol Microbiol. (2010) 60:2735–8. 10.1099/ijs.0.017590-0 20061495

[40] Wang Q Zhang Y Zheng N Guo L Song X Zhao S. Biological system responses of dairy cows to aflatoxin B1 exposure revealed with metabolomic changes in multiple biofluids. Toxins. (2019) 11:77. 10.3390/toxins11020077 30717092 PMC6410036

[41] Abarghuei MJ Rouzbehan Y Salem AZM Zamiri MJ. Nitrogen balance, blood metabolites and milk fatty acid composition of dairy cows fed pomegranate-peel extract. Livestock Sci. (2014) 164:72–80. 10.1016/j.livsci.2014.03.021

[42] Tsunoda E Gross JJ Kawashima C Bruckmaier RM Kida K Miyamoto A. Feed-derived volatile basic nitrogen increases reactive oxygen species production of blood leukocytes in lactating dairy cows: poor fermented silage and cow metabolism. Anim Sci J. (2017) 88:125–33. 10.1111/asj.12608 27145971

[43] Kulka M Bełtowski J Kluciński W Orłowska M Kołodziejska J Kleczkowski M. Serum paraoxonase-1 activity of dairy Holstein-Fresian cows in different lactation stages – preliminary study. Polish J Vet Sci. (2014) 17:143–7. 10.2478/pjvs-2014-0019 24724482

[44] Silper BF Lana AMQ Carvalho AU Ferreira CS Franzoni APS Lima JAM. Effects of milk replacer feeding strategies on performance, ruminal development, and metabolism of dairy calves. J Dairy Sci. (2014) 97:1016–25. 10.3168/jds.2013-7201 24342682

[45] Daniels KM Hill SR Knowlton KF James RE McGilliard ML Akers RM. Effects of milk replacer composition on selected blood metabolites and hormones in preweaned holstein heifers. J Dairy Sci. (2008) 91:2628–40. 10.3168/jds.2007-0859 18565922

[46] Harzia H Ilves A Ots M Henno M Jõudu I Kaart T. Alterations in milk metabolome and coagulation ability during the lactation of dairy cows. J Dairy Sci. (2013) 96:6440–8. 10.3168/jds.2013-6808 23958001

[47] Scano P Murgia A Pirisi FM Caboni P. A gas chromatography-mass spectrometry-based metabolomic approach for the characterization of goat milk compared with cow milk. J Dairy Sci. (2014) 97:6057–66. 10.3168/jds.2014-8247 25108860

[48] Klein MS Buttchereit N Miemczyk SP Immervoll A-K Louis C Wiedemann S. NMR metabolomic analysis of dairy cows reveals milk glycerophosphocholine to phosphocholine ratio as prognostic biomarker for risk of ketosis. J Proteome Res. (2012) 11:1373–81. 10.1021/pr201017n 22098372

[49] Saleem F Ametaj BN Bouatra S Mandal R Zebeli Q Dunn SM. A metabolomics approach to uncover the effects of grain diets on rumen health in dairy cows. J Dairy Sci. (2012) 95:6606–23. 10.3168/jds.2012-5403 22959937

[50] Hailemariam D Mandal R Saleem F Dunn SM Wishart DS Ametaj BN. Identification of predictive biomarkers of disease state in transition dairy cows. J Dairy Sci. (2014) 97:2680–93. 10.3168/jds.2013-6803 24630653

[51] Sundekilde UK Gustavsson F Poulsen NA Glantz M Paulsson M Larsen LB. Association between the bovine milk metabolome and rennet-induced coagulation properties of milk. J Dairy Sci. (2014) 97:6076–84. 10.3168/jds.2014-8304 25087032

[52] Zhang H Tong J Zhang Y Xiong B Jiang L. Metabolomics reveals potential biomarkers in the rumen fluid of dairy cows with different levels of milk production. Asian-Australas J Anim Sci. (2020) 33:79–90. 10.5713/ajas.19.0214 31480145 PMC6946990

[53] National Research Council (U.S.) ed. Nutrient Requirements of Dairy Cattle. 7th rev. ed. Washington, DC: National Academy Press (2001).

[54] Contreras GA Mattmiller SA Raphael W Gandy JC Sordillo LM. Enhanced n-3 phospholipid content reduces inflammatory responses in bovine endothelial cells. J Dairy Sci. (2012) 95:7137–50. 10.3168/jds.2012-5729 23040031

[55] Bertram HC Yde CC Zhang X Kristensen NB. Effect of dietary nitrogen content on the urine metabolite profile of dairy cows assessed by Nuclear Magnetic Resonance (NMR)-Based Metabolomics. J Agric Food Chem. (2011) 59:12499–505. 10.1021/jf204201f 22059599

[56] Lan W Ren Y Wang Z Liu J Liu H. Metabolic profile reveals the immunosuppressive mechanisms of methionyl-methionine in lipopolysaccharide-induced inflammation in bovine mammary epithelial cell. Animals. (2021) 11:833. 10.3390/ani11030833 33809487 PMC8000761

[57] Waisbren SE Noel K Fahrbach K Cella C Frame D Dorenbaum A. Phenylalanine blood levels and clinical outcomes in phenylketonuria: a systematic literature review and meta-analysis. Mol Genet Metab. (2007) 92:63–70. 10.1016/j.ymgme.2007.05.006 17591452

[58] Lemmon MA Schlessinger J. Cell signaling by receptor tyrosine kinases. Cell. (2010) 141:1117–34. 10.1016/j.cell.2010.06.011 20602996 PMC2914105

[59] Garcia-Lino AM Gomez-Gomez A Garcia-Mateos D de la Fuente A Alvarez AI Pozo OJ. Analysis of the interaction between tryptophan-related compounds and ATP-binding cassette transporter G2 (ABCG2) using targeted metabolomics. Food Chem. (2021) 344:128665. 10.1016/j.foodchem.2020.128665 33250293

