# Supplementary material for: Metabolomics Analysis Across Multiple Biofluids Reveals the Metabolic Responses of Lactating Holstein Dairy Cows to Fermented Soybean Meal Replacement
Source: Front Vet Sci. 2022 May 13;9:812373. doi: 10.3389/fvets.2022.812373 (PMC9136663; doi:10.3389/fvets.2022.812373)
Supplement: Supplementary file 1 [file Table_1.PDF]

Supplementary Material

Metabolomics analysis across multiple biofluids reveals the metabolic responses of lactating Holstein dairy cows to fermented soybean meal replacement

Zuo Wang [1], Yuannian Yu [1,4], Weijun Shen [1], Zhiliang Tan [2], Shaoxun Tang [2]*, Hui Yao [3], Jianhua He [1]* and Fachun Wan [1]*

[1] College of Animal Science and Technology, Hunan Agricultural University, Changsha, Hunan 410128, China

[2] CAS Key Laboratory of Agro-Ecological Processes in Subtropical Region, National Engineering Laboratory for Pollution Control and Waste Utilization in Livestock and Poultry Production, Hunan Provincial Key Laboratory of Animal Nutrition & Physiology and Metabolism, Institute of Subtropical Agriculture, Chinese Academy of Sciences, Changsha, Hunan 410125, China

[3] Nanshan Dairy Co. Ltd., Shaoyang, Hunan 422500, China

[4] Rudong Agriculture Bureau, Nantong, Jiangsu 226400, China

*** Correspondence:**

Fachun Wan; Jianhua He; Shaoxun Tang

wanfc@sina.com; 895732301@qq.com; shaoxuntang@163.com

Supplementary Tables

Table S1. The nutritional compositions and amino acids profiles of SBM and FSBM

Nutrient composition, % DM	SBM[1]	FSBM[2]
Organic matter	93.94	92.64
Crude protein	49.71	55.04
Ether extract	1.42	4.49
Neutral detergent fiber	15.22	24.41
Acid detergent fiber	0.08	0.05
Ca	0.22	0.34
P	0.67	0.71
Aspartate	0.79	0.85
Threonine	0.43	0.45
Serine	0.35	0.55
Glutamate	1.07	2.15
Glycine	0.48	0.58
Alanine	0.63	0.89
Valine	0.50	0.65
Methionine	0.06	0.07
Isoleucine	0.40	0.43
Leucine	0.66	1.18
Tryptophan	0.24	0.33
Phenylalanine	0.44	0.54
Lysine	0.59	0.54
Histidine	0.18	0.40
Arginine	0.31	0.76
Proline	0.42	1.00

[1] SBM = soybean meal; [2] FSBM = fermented soybean meal.
