# Supplementary material for: Metabolomics Analysis Across Multiple Biofluids Reveals the Metabolic Responses of Lactating Holstein Dairy Cows to Fermented Soybean Meal Replacement
Source: Front Vet Sci. 2022 May 13;9:812373. doi: 10.3389/fvets.2022.812373 (PMC9136663; doi:10.3389/fvets.2022.812373)
Supplement: Supplementary file 4 [file Table_4.PDF]

Supplementary Material

Metabolomics analysis across multiple biofluids reveals the metabolic responses of lactating Holstein dairy cows to fermented soybean meal replacement

Zuo Wang [1], Yuannian Yu [1,4], Weijun Shen [1], Zhiliang Tan [2], Shaoxun Tang [2]*, Hui Yao [3], Jianhua He [1]* and Fachun Wan [1]*

[1] College of Animal Science and Technology, Hunan Agricultural University, Changsha, Hunan 410128, China

[2] CAS Key Laboratory of Agro-Ecological Processes in Subtropical Region, National Engineering Laboratory for Pollution Control and Waste Utilization in Livestock and Poultry Production, Hunan Provincial Key Laboratory of Animal Nutrition & Physiology and Metabolism, Institute of Subtropical Agriculture, Chinese Academy of Sciences, Changsha, Hunan 410125, China

[3] Nanshan Dairy Co. Ltd., Shaoyang, Hunan 422500, China

[4] Rudong Agriculture Bureau, Nantong, Jiangsu 226400, China

*** Correspondence:**

Fachun Wan; Jianhua He; Shaoxun Tang

wanfc@sina.com; 895732301@qq.com; shaoxuntang@163.com

Supplementary Tables

Table S3. Classification and analysis of significantly different metabolites in rumen liquid, plasma, milk, and urine between the SBM group and FSBM group

Metabolite	Sub Class[1]	RT[2]	m/z[3]	VIP[4]	*P*-value	FC[5]
Rumen liquid						
p-Octopamine	1-hydroxy-2-unsubstituted benzenoids	48.52	154.09	1.736	0.050	1.511
Pyruvic acid	Alpha-keto acids and derivatives	79.29	87.01	2.360	0.006	1.736
Glutaminylproline	Amino acids, peptides, and analogues	333.13	244.13	2.857	0.000	2.024
Spinacine	Amino acids, peptides, and analogues	238.19	168.08	2.902	0.000	1.570
Ophthalmic acid	Amino acids, peptides, and analogues	404.97	290.13	2.223	0.018	1.440
3-Methoxytyrosine	Amino acids, peptides, and analogues	389.92	212.09	1.713	0.049	1.319
Clavulanate	Amino acids, peptides, and analogues	49.23	198.04	2.390	0.007	1.273
L-Isoleucine	Amino acids, peptides, and analogues	308.16	132.10	2.107	0.036	1.228
Streptozocin	Carbohydrates and carbohydrate conjugates	246.99	266.10	2.465	0.007	1.700
11(R)-HETE	Eicosanoids	59.81	319.23	2.706	0.000	1.349
L-Acetylcarnitine	Fatty acid esters	392.89	204.12	2.156	0.016	1.375
3-Methyladipic acid	Fatty acids and conjugates	193.75	159.07	2.110	0.016	1.889
12,13-DHOME	Fatty acids and conjugates	59.71	315.25	2.077	0.029	1.204
Epoxymurin B	Fatty alcohols	161.50	531.48	2.788	0.000	2.113
9-Oxoasimicinone	Fatty alcohols	174.55	637.46	2.117	0.033	1.923
Cohibin A	Fatty alcohols	169.32	549.49	2.042	0.037	1.378
1-O-Hexadecyl-2-O-dihomogammalinolenoylglycero-3-phosphocholine	Glycerophosphocholines	81.59	770.61	1.946	0.032	1.322
PE(14:1(9Z)/18:0)	Glycerophosphoethanolamines	170.17	690.50	2.497	0.018	1.540
PE(18:1(11Z)/14:1(9Z))	Glycerophosphoethanolamines	169.30	688.49	2.563	0.002	1.483
PE(P-18:1(11Z)/14:1(9Z))	Glycerophosphoethanolamines	161.53	672.49	2.290	0.007	1.379
PE(P-18:1(11Z)/14:0)	Glycerophosphoethanolamines	163.27	674.51	1.951	0.038	1.237
Indole-3-carbinol	Indoles	232.85	146.06	2.516	0.004	1.251

1,2-Dihydro-1,1,6-trimethylnaphthalene	Naphthalenes (Class)	31.73	173.13	2.462	0.006	1.194
Pyridoxamine	Pyridoxamines	63.67	169.10	1.862	0.048	1.245
Butalbital	Pyrimidines and pyrimidine derivatives	377.25	225.12	2.214	0.017	1.489
Kynurenic acid	Quinoline carboxylic acids	240.73	188.03	2.689	0.000	1.721
Lucanthone	Thiochromenes (Class)	332.88	341.17	1.989	0.028	1.347
Glycyrrhetinic acid	Triterpenoids	46.56	469.33	1.903	0.018	1.774
Chlorogenic acid	Alcohols and polyols	124.64	353.09	2.023	0.026	0.771
Formiminoglutamic acid	Amino acids, peptides, and analogues	414.57	175.07	2.337	0.005	0.649
1,2-Dihydroxy-3-keto-5-methylthiopentene	Carbonyl compounds	123.41	163.04	2.270	0.015	0.789
N-Acetylhistamine	Carboxylic acid derivatives	146.53	154.10	1.866	0.040	0.676
2-(3,4-Dihydroxybenzoyloxy)-4,6-dihydroxybenzoate	Depsides and depsidones (Class)	229.38	307.04	2.369	0.003	0.594
Imidazole-4-acetaldehyde	Imidazoles	61.01	111.06	2.264	0.010	0.788
Nabumetone	Naphthalenes (Class)	169.24	227.11	1.898	0.048	0.765
Inosine	Purine nucleosides (Class)	228.88	267.07	2.382	0.004	0.489
Arabinosylhypoxanthine	Purine nucleosides (Class)	229.38	269.09	2.347	0.004	0.480
Adenosine	Purine nucleosides (Class)	205.79	268.10	1.967	0.002	0.444
(±)-Rollipyrrole	Pyrrolines (Class)	219.54	289.15	1.892	0.039	0.639
Doxycycline	Tetracyclines (Class)	128.17	443.15	1.967	0.032	0.802
Plasma						
Pantothenic acid	Alcohols and polyols	283.88	218.10	2.434	0.004	1.261
6beta-Hydroxytestosterone	Androstane steroids	30.61	303.20	2.197	0.029	1.240
8-Isoprostaglandin E1	Eicosanoids	52.16	353.23	2.068	0.025	1.360
19-Hydroxy-PGE2	Eicosanoids	52.94	367.22	2.206	0.027	1.342
2-Hydroxystearic acid	Fatty acids and conjugates	49.53	299.26	2.115	0.023	1.163
lysoPC(28:0)	Glycerophosphocholines	171.67	664.53	1.963	0.037	1.549

LysoPE(20:5(5Z,8Z,11Z,14Z,17Z)/0:0)	Glycerophosphoethanolamines	223.50	500.27	1.808	0.039	1.223
1,2,3,4,Tetrahydro-1,5,7-trimethylnapthalene	Tetralins (Class)	31.27	175.15	2.192	0.018	1.366
Indoxyl sulfate	Arylsulfates	27.22	212.00	1.962	0.033	0.817
Epidermin	Carbohydrates and carbohydrate conjugates	416.06	262.13	2.136	0.033	0.731
Ethyl tetradecanoate	Fatty acid esters	67.97	255.23	2.464	0.010	0.803
2-Hydroxymyristic acid	Fatty acids and conjugates	53.99	243.20	2.394	0.016	0.763
Pirbuterol	Hydroxypyridines	389.96	241.15	2.350	0.043	0.791
3-(4-hydroxyphenyl)-3,4-dihydro-2H-1-benzopyran-7-ol	Isoflavans	25.05	243.10	2.841	0.000	0.425
Cytidine	Pyrimidine nucleosides (Class)	254.95	244.09	2.199	0.020	0.719
Cytosine	Pyrimidines and pyrimidine derivatives	254.97	112.05	2.305	0.014	0.722
Milk						
Trigonelline	Alkaloids and derivatives (Super Class)	460.85	138.05	1.817	0.034	3.213
benzene-1,2,4-triol	Benzenetriols and derivatives	414.58	125.02	1.866	0.032	1.133
Galactose 1-phosphate	Carbohydrates and carbohydrate conjugates	483.22	259.02	2.004	0.043	7.539
N-Acetyl-glucosamine 1-phosphate	Carbohydrates and carbohydrate conjugates	461.74	324.04	1.564	0.047	2.569
Maltotriose	Carbohydrates and carbohydrate conjugates	414.55	503.16	1.771	0.047	1.116
Beta-D-Galactose	Carbohydrates and carbohydrate conjugates	414.58	179.06	1.789	0.040	1.112
Trehalose 6-phosphate	Carbohydrates and carbohydrate conjugates	497.88	421.08	1.782	0.036	1.544
D-Xylulose	Carbohydrates and carbohydrate conjugates	415.41	149.04	1.764	0.033	1.191
2-Dodecanone	Carbonyl compounds	29.84	285.24	1.802	0.027	1.269
Butyrylcarnitine	Fatty acid esters	315.79	232.15	1.790	0.028	1.413
Diethyl fumarate	Fatty acid esters	278.50	173.08	1.716	0.040	1.377
Propionylcarnitine	Fatty acid esters	298.19	218.14	2.227	0.004	1.272
Methylglutaric acid	Fatty acids and conjugates	418.92	145.05	1.608	0.027	1.477
cis,cis-Muconic acid	Fatty acids and conjugates	414.59	141.02	2.015	0.012	1.246

3-Hexenedioic acid	Fatty acids and conjugates	435.31	145.05	2.259	0.003	1.197
Diepomuricanin A	Fatty alcohols	169.50	547.47	1.937	0.042	1.538
PE(16:0/20:4(5Z,8Z,11Z,14Z))	Glycerophosphoethanolamines	163.46	740.52	1.992	0.004	1.624
PE(18:2(9Z,12Z)/15:0)	Glycerophosphoethanolamines	168.66	702.50	1.803	0.031	1.500
PE(16:0/18:2(9Z,12Z))	Glycerophosphoethanolamines	167.74	716.52	2.116	0.009	1.331
PS(18:2(9Z,12Z)/18:1(9Z))	Glycerophosphoserines	219.20	786.53	1.746	0.042	1.252
PS(20:1(11Z)/15:0)	Glycerophosphoserines	220.16	776.54	1.936	0.023	1.200
PS(18:2(9Z,12Z)/16:0)	Glycerophosphoserines	220.90	760.51	1.609	0.038	1.411
Ethyl glucuronide	Organooxygen compounds	414.58	221.07	1.901	0.029	1.128
Pregnanetriol	Pregnane steroids	29.84	337.27	1.775	0.036	1.336
Adenosine	Purine nucleosides (Class)	177.43	268.10	1.863	0.032	1.397
Maltol	Pyranones and derivatives	460.68	127.04	2.325	0.002	1.497
L-2-Amino-3-methylenehexanoic acid	Amino acids, peptides, and analogues	375.29	144.10	1.966	0.018	0.843
L-Cyclo(alanylglycyl)	Amino acids, peptides, and analogues	378.89	129.07	1.778	0.034	0.815
Butyl 2-aminobenzoate	Benzoic acids and derivatives	218.23	194.12	1.715	0.048	0.894
(R)-2-Hydroxy-2H-1,4-benzoxazin-3(4H)-one	Benzoxazinones	18.98	166.05	2.106	0.023	0.781
Aminofructose 6-phosphate	Carbohydrates and carbohydrate conjugates	282.19	260.05	1.950	0.032	0.592
D-2,3-Dihydroxypropanoic acid	Carbohydrates and carbohydrate conjugates	336.17	105.02	1.806	0.044	0.799
N-Acetyl-D-glucosamine	Carbohydrates and carbohydrate conjugates	267.05	220.08	1.801	0.020	0.702
Hypogeic acid	Fatty acids and conjugates	40.34	253.22	1.896	0.011	0.777
Tridecanoic acid	Fatty acids and conjugates	36.21	213.19	1.770	0.036	0.770
Artemoin A	Fatty alcohols	169.49	551.50	2.319	0.000	0.564
Glucosylceramide (d18:1/22:0)	Glycosphingolipids	42.28	784.66	1.784	0.048	0.720
6-Hydroxy-1H-indole-3-acetamide	Hydroxyindoles	35.92	191.08	1.887	0.026	0.699
Indoleacetaldehyde	Indoles	32.76	158.06	1.694	0.025	0.516

13S-hydroxyoctadecadienoic acid	Lineolic acids and derivatives	47.11	295.23	1.958	0.016	0.587
(10E,12Z)-(9S)-9-Hydroperoxyoctadeca-10,12-dienoic acid	Lineolic acids and derivatives	57.14	311.22	2.020	0.039	0.554
Nicotinic acid mononucleotide	Nicotinic acid nucleotides	354.54	256.08	1.907	0.021	0.584
Choline	Quaternary ammonium salts	289.19	104.11	1.893	0.047	0.902
Urine						
Dimethylethanolamine	Amines	281.28	90.09	1.839	0.006	1.199
Isoleucyl-Alanine	Amino acids, peptides, and analogues	360.41	203.14	1.719	0.012	1.446
Hydroxyprolyl-Leucine	Amino acids, peptides, and analogues	358.28	245.15	1.784	0.019	1.513
Norvaline	Amino acids, peptides, and analogues	332.20	118.09	1.256	0.028	1.276
p-Cresol sulfate	Arylsulfates	25.06	187.01	1.764	0.023	1.530
Methyl 2-aminobenzoate	Benzoic acids and derivatives	26.16	152.07	1.717	0.008	1.327
Aztreonam	Beta lactams	247.43	436.06	1.543	0.028	1.796
Netilmicin	Carbohydrates and carbohydrate conjugates	83.30	476.31	1.357	0.042	1.905
HMBOA-Glc	Carbohydrates and carbohydrate conjugates	218.38	358.11	1.584	0.032	1.664
6-(Methylthio)hexyl glucosinolate	Carbohydrates and carbohydrate conjugates	215.44	450.10	1.951	0.000	1.598
Aldehydo-D-xylose	Carbohydrates and carbohydrate conjugates	151.36	149.05	1.458	0.019	1.469
Alpha-Lactose	Carbohydrates and carbohydrate conjugates	151.36	341.11	1.430	0.023	1.409
N-Acetylputrescine	Carboximidic acids	351.84	131.12	1.875	0.016	2.819
15-Methylpalmitate	Fatty acids and conjugates	3.68	269.25	1.672	0.015	3.404
1-(beta-D-Glucopyranosyloxy)-3-octanone	Fatty acyl glycosides	269.91	307.17	1.620	0.012	2.488
7-hydroxy-2-phenyl-4H-chromen-4-one	Flavones	22.67	239.07	1.624	0.013	1.195
Bergapten	Furanocoumarins	23.18	215.04	1.960	0.005	1.885
Glycerylphosphorylethanolamine	Glycerophosphoethanolamines	203.85	216.06	1.233	0.045	1.376
Pentostatin	Imidazodiazepines (Class)	328.82	269.12	1.445	0.037	1.185
1,3-Dihydro-(2H)-indol-2-one	Indolines	28.69	134.06	1.771	0.014	1.326

Nabumetone	Naphthalenes (Class)	175.00	227.11	1.369	0.045	1.338
Nitrite	Non-metal nitrites	358.12	118.09	1.843	0.024	1.775
Cerulenin	Oxirane carboxylic acids and derivatives	183.57	222.11	1.902	0.003	1.571
Nelarabine	Purine nucleosides (Class)	210.77	296.10	1.048	0.028	2.403
N6-Methyladenosine	Purine nucleosides (Class)	303.98	282.12	1.801	0.020	1.253
3-Methyladenine	Purines and purine derivatives	303.98	150.08	1.838	0.015	1.217
FAPy-adenine	Pyrimidines and pyrimidine derivatives	184.01	154.07	1.692	0.018	1.233
Quinoline	Quinolines and derivatives (Class)	49.11	130.07	1.683	0.019	1.355
p-Octopamine	1-hydroxy-2-unsubstituted benzenoids	28.82	154.09	1.993	0.002	0.491
p-Hydroxymandelic acid	1-hydroxy-2-unsubstituted benzenoids	208.32	167.03	1.834	0.001	0.450
2-(2-Furanyl)piperidine	Amines	215.72	152.11	1.286	0.047	0.787
N-Acetylleucine	Amino acids, peptides, and analogues	80.65	174.11	1.423	0.021	0.781
Prolyl-Alanine	Amino acids, peptides, and analogues	82.76	187.11	1.376	0.022	0.752
N-Acetyl-L-alanine	Amino acids, peptides, and analogues	275.54	130.05	1.569	0.034	0.737
Tiglylglycine	Amino acids, peptides, and analogues	218.75	156.07	1.531	0.010	0.736
N-Acetylglutamine	Amino acids, peptides, and analogues	326.16	189.09	1.580	0.042	0.728
Tryptophyl-Tryptophan	Amino acids, peptides, and analogues	234.29	391.17	2.089	0.000	0.588
Pipecolic acid	Amino acids, peptides, and analogues	289.08	130.09	1.369	0.043	0.586
2-Furoylglycine	Amino acids, peptides, and analogues	219.22	168.03	1.728	0.017	0.561
3-Ethylidenehexahydropyrrolo[1,2-a]pyrazine-1,4-dione	Amino acids, peptides, and analogues	122.48	181.10	1.558	0.027	0.544
Propionylglycine	Amino acids, peptides, and analogues	259.26	130.05	1.809	0.028	0.527
D-Proline	Amino acids, peptides, and analogues	74.62	116.07	1.921	0.005	0.503
L-Tyrosine	Amino acids, peptides, and analogues	322.04	180.07	1.492	0.023	0.471
Methionine sulfoxide	Amino acids, peptides, and analogues	254.22	164.04	1.565	0.011	0.436
2,3,4,5-Tetrahydropiperidine-2-carboxylate	Amino acids, peptides, and analogues	129.28	126.06	1.418	0.044	0.361

D-4'-Phosphopantothenate	Amino acids, peptides, and analogues	330.40	300.08	1.979	0.005	0.348
Cinnamoylglycine	Amino acids, peptides, and analogues	202.48	204.07	1.355	0.019	0.810
N-Methyl-D-aspartic acid	Amino acids, peptides, and analogues	279.98	146.05	1.393	0.036	0.733
N-Acetylhistidine	Amino acids, peptides, and analogues	331.39	198.09	2.072	0.000	0.708
L-Glutamic acid	Amino acids, peptides, and analogues	414.38	146.05	1.643	0.024	0.505
Isoproterenol	Benzenediols	59.50	210.11	2.153	0.005	0.354
3-Hydroxyanthranilic acid	Benzoic acids and derivatives	163.38	152.03	1.538	0.023	0.726
Butyl 2-aminobenzoate	Benzoic acids and derivatives	218.61	194.12	2.016	0.001	0.545
N,N'-diacetylchitobiose	Carbohydrates and carbohydrate conjugates	254.14	425.18	1.334	0.022	0.619
Mycotoxin T 2	Carbohydrates and carbohydrate conjugates	240.26	342.15	1.949	0.001	0.348
Dopamine glucuronide	Carbohydrates and carbohydrate conjugates	321.60	330.12	1.951	0.001	0.331
2,8-Dihydroxyquinoline-beta-D-glucuronide	Carbohydrates and carbohydrate conjugates	334.41	338.09	1.999	0.011	0.536
Acetamidopropanal	Carbonyl compounds	97.23	116.07	1.299	0.026	0.783
2-Acetyl-3,6-dimethylpyrazine	Carbonyl compounds	72.90	151.09	1.594	0.015	0.763
2',4'-Dihydroxyacetophenone	Carbonyl compounds	95.44	151.04	1.598	0.021	0.637
N-Acetylhistamine	Carboxylic acid derivatives	206.79	154.10	1.625	0.005	0.676
N-Acetyl-beta-alanine	Carboxylic acids	299.20	130.05	1.600	0.040	0.702
Maleic acid	Dicarboxylic acids and derivatives	45.56	115.00	1.823	0.003	0.665
8-Isoprostaglandin E1	Eicosanoids	92.34	353.23	1.382	0.038	0.635
Thromboxane B3	Eicosanoids	182.10	367.21	1.339	0.016	0.612
Tetradecanedioic acid	Fatty acids and conjugates	236.17	257.18	1.881	0.000	0.580
Caproic acid	Fatty acids and conjugates	198.12	115.08	1.166	0.030	0.183
Azelaic acid	Fatty acids and conjugates	357.87	187.10	1.314	0.040	0.829
Hexadecanedioic acid	Fatty acids and conjugates	90.68	285.21	1.858	0.002	0.606
3-Hydroxyvaleric acid	Fatty acids and conjugates	244.42	117.06	1.757	0.027	0.543

Traumatic acid	Fatty acids and conjugates	223.20	227.13	2.185	0.000	0.526
Dodecanedioic acid	Fatty acids and conjugates	297.69	229.14	1.654	0.014	0.543
Corchoionoside B	Fatty acyl glycosides	322.49	401.18	1.627	0.040	0.582
Sudachiin A	Flavonoid glycosides	238.03	523.15	1.294	0.029	0.369
4-Hydroxycinnamic acid	Hydroxycinnamic acids and derivatives	128.63	163.04	1.596	0.008	0.648
Indole-3-carboxylic acid	Indolecarboxylic acids and derivatives	335.06	160.04	1.999	0.012	0.516
Tryptophanol	Indoles	202.49	160.08	1.317	0.028	0.810
Indoleacetic acid	Indolyl carboxylic acids and derivatives	207.01	176.07	1.457	0.038	0.765
5-Hydroxyindoleacetic acid	Indolyl carboxylic acids and derivatives	186.99	190.05	1.524	0.010	0.545
8-8'-Dehydrodiferulic acid	Lignans, neolignans and related compounds (Super Class)	253.32	387.10	1.368	0.025	0.524
Jasmonic acid	Lineolic acids and derivatives	74.32	209.12	1.553	0.042	0.813
Baicalin	Macrolides and analogues (Class)	279.15	445.08	1.800	0.000	0.199
3-Hydroxycapric acid	Medium-chain hydroxy acids and derivatives	117.55	187.13	1.654	0.015	0.504
Vanillylamine	Methoxyphenols	86.77	154.09	1.341	0.039	0.679
Isobornyl propionate	Monoterpenoids	299.60	144.10	1.703	0.012	0.586
Polyvidone	N-alkylpyrrolidines	331.78	178.05	1.195	0.046	0.800
2-(1-Pyrrolidinyl)-3-pentanone	N-alkylpyrrolidines	111.43	156.14	1.405	0.035	0.598
O-Desmethylnaproxen	Naphthols and derivatives	252.26	215.07	1.921	0.039	0.290
4-Nitrophenol	Nitrophenols	319.46	138.02	1.494	0.036	0.772
2-Aminonaphthalene	Not Available	203.74	144.08	1.671	0.005	0.688
Gemfibrozil	Phenol ethers(Class)	91.51	249.15	2.170	0.000	0.515
Metaraminol	Phenylpropanes	247.42	168.10	2.053	0.000	0.449
3-(3-Hydroxyphenyl)propanoic acid	Phenylpropanoic acids (Class)	161.89	165.06	1.703	0.013	0.588
3-(2-Hydroxyphenyl)propanoic acid	Phenylpropanoic acids (Class)	64.69	165.06	1.604	0.047	0.675

Phensuximide	Phenylpyrrolidines	197.79	190.09	1.492	0.021	0.504
Phenylpyruvic acid	Phenylpyruvic acid derivative	181.85	163.04	1.904	0.004	0.517
Ziprasidone	Piperazines	260.03	413.12	1.518	0.041	0.543
Ketoconazole	Piperazines	256.92	87.09	1.665	0.022	0.380
Cytokinin B	Purines and purine derivatives	396.89	226.11	1.629	0.005	0.413
Pyridoxal	Pyridine carboxaldehydes	214.15	168.07	1.736	0.014	0.708
Secobarbital	Pyrimidines and pyrimidine derivatives	273.25	237.12	1.416	0.040	0.699
Pyrrole-2-carboxylic acid	Pyrrole carboxylic acids and derivatives	61.39	110.02	1.662	0.015	0.578
2-Pyrrolidinone	Pyrrolidones	119.15	86.06	1.532	0.033	0.776
Pterolactam	Pyrrolidones	55.03	116.07	1.595	0.007	0.682
7C-aglycone	Quinone and hydroquinone lipids	24.43	299.13	1.662	0.015	0.674
3-Methyl-2-oxovaleric acid	Short-chain keto acids and derivatives	137.57	129.06	1.611	0.035	0.801
2-Ketobutyric acid	Short-chain keto acids and derivatives	94.00	101.02	1.871	0.006	0.139
Lippioside I	Terpene glycosides	283.87	539.18	1.628	0.012	0.582
Cynaroside A	Terpene lactones	330.38	445.20	1.779	0.006	0.398
Enterolactone	Tetrahydrofuran lignans	210.27	299.13	1.462	0.017	0.345
N-Methyltryptamine	Tryptamines and derivatives	308.48	175.12	1.508	0.014	0.594

[1] Classification at the level of Sub Class for the metabolite based on the HMDB database, and the classification at the level of Class or Super Class is offered with a remark if the information at the Sub Class level or Class level is not available in the HMDB database; [2] RT = retention time; [3] m/z = the ratio of mass to charge of the metabolite; [4] VIP = variable importance in the projection; [5] FC = fold change, the ratio of the quantity of the metabolite in the FSBM group to that in the SBM group. It means that the amount of the metabolite is higher in the FSBM treatment than that in the SBM treatment, when the corresponding FC value is above 1.
